# Comprehensive analysis of the prognosis and immune infiltrates for the BET protein family reveals the significance of BRD4 in glioblastoma multiforme

**DOI:** 10.3389/fcell.2023.1042490

**Published:** 2023-01-12

**Authors:** Yintao Ye, Wei Zhong, Junqiang Qian, Jie Zhang, Tingting Xu, Ruyi Han, Jiangeng Han, Chunwei Wang, Lichao Song, Xianwei Zeng, Hong Wang

**Affiliations:** ^1^ Department of Pharmacy, Tianjin Medical University Cancer Institute and Hospital, National Clinical Research Center for Cancer, Tianjin^’^s Clinical Research Center for Cancer, Key Laboratory of Cancer Immunology and Biotherapy, Tianjin, China; ^2^ Department of quality, Tianjin Plastics Research Institute Co., Ltd, Tianjin, China; ^3^ Geriatric Health Engineering Research Center, Institute of Biomedical Engineering, Chinese Academy of Medical Science and Peking Union Medical College, Tianjin, China; ^4^ Rehabilitation hospital affiliated to National Research Center for Rehabilitation Technical Aids, Beijing, China

**Keywords:** glioblastoma multiforme, BET protein family, prognosis, immune checkpoint, immune infiltrates

## Abstract

**Background:** Glioblastoma multiforme (GBM) is the most common and invasive primary central nervous system tumor. The prognosis after surgery, radiation and chemotherapy is very poor. Bromodomain (BRD) proteins have been identified in oncogenic rearrangements, and play a key role in the development of multiple cancers. However, the relationship between BET proteins and prognosis of GBM are still worth exploring, and the distinct functions of BET proteins and tumor immunology in GBM have not been fully elucidated. Therefore, it is particularly important to develop effective biomarkers to predict the prognosis of GBM patients.

**Methods:** Metascape, David, Kaplan-Meier Plotter, Oncomine, GEPIA, TCGA, TIMER, and LinkedOmics databases were used to assess the expression and prognosis for BET proteins in GBM. ROC analysis of risk model was established to identify the correlation between BET genes and overall survival in GBM patients. TIMER and GEPIA databases were used to comprehensively investigate the correlation between BET genes and tumor immune infiltration cells. Moreover, the image of immunohistochemistry staining of BET proteins in normal tissue and tumor tissue were retrived from the HPA database. In addition, differential analysis and pathway enrichment analysis of BRD4 gene expression profile were also carried out. Finally, immune-fluorescence and Western blot were used to clarify the expression of BRD4 in GBM cells.

**Results:** Bioinformatics analysis showed that the expression levels of BET genes in GBM may play an important role in oncogenesis. Specifically, bioinformatic and immunohistochemistry analysis showed that BRD4 protein was more highly expressed in tumor tissues than that in normal tissues. The high expression of BRD4 was associated with poor prognosis in GBM. The expression of BET genes were closely related to the immune checkpoint in GBM. The correlation effect of BRD4 was significantly higher than other BET genes, which represented negative correlation with immune checkpoint. The expression of BRD4 was positively associated with tumor purity, and negatively associated with immune infiltration abundance of macrophage, neutrophil and CD8^+^ T-cell, respectively. Cox analysis showed that the model had good survival prediction and prognosis discrimination ability. In addition, the expression levels of BRD4 protein was significantly higher in U-251 MG cells than that in normal cells, which was consistent with the results of bioinformatics data.

**Conclusion:** This study implied that BRD4 could be hopeful prognostic biomarker in GBM. The increased expression of BRD4 may act as a molecular marker to identify GBM patients with high-risk subgroups. BRD4 may be a valuable prognostic biomarker, and a potential target of precision therapy against GBM.

## Introduction

Gliomas are the most important tumors in the field of neurooncology. Malignant gliomas account for about 70% of all gliomas, the overall 5-year relative survival rate was almost 32.1% (Lapointe et al., 2018; Barnholtz-Sloan et al., 2018). Glioblastoma multiforme (GBM) is a primary malignant brain tumor. Because these tumors are so malignant, most patients have a poor prognosis after surgery, radiation, and chemotherapy (Wu et al., 2021). GBM is a World Health Organization (WHO) grade IV glioma with the worst prognosis (Wesseling and Capper, 2018). Despite the active treatment efforts and various combination treatment schemes, the prognosis of GBM patients is still very poor. The OS is 10.2 months, and the 5-year survival rate is less than 5% (Gao et al., 2022; Li et al., 2022). GBM is also the big challenges in cancer treatment. Therefore, it is particularly important to understand the molecular mechanism of GBM and develop the appropriate and effective biomarkers or therapeutic targets to predict the prognosis of patients with GBM. Tumour associated macrophages (TAMs) are the largest immune cell population found in GBM, accounting for 30%–40% of tumor cells (Charles et al., 2012). TAMs directly inhibit T-cell function through the surface presentation of PD-L1, which activates PD-1 and CTLA-4, respectively (Niedbała et al., 2022). Immunotherapy is a revolution in cancer treatment (Ballas, 2018). Therefore, the highly immunosuppressive property of GBM microenvironment is a new strategy for developing therapeutic schemes.

Bromodomain and extra-terminal domain (BET) belongs to Bromodomain protein family members mainly including BRD2, BRD3, BRD4 and BRDT. BET protein family is involved in recognition of histone lysine acetylation (Donati et al., 2018; Pervaiz et al., 2018). BET proteins are important epigenetic regulators and transcriptional regulators, which play an important role in cell cycle (Gao et al., 2018). Additionally, BET proteins have been identified in carcinogenic rearrangements, which is a fusion protein leading to high carcinogenicity (Shi and Vakoc, 2014). Meanwhile, a variety of small molecule inhibitors of BET proteins have also been developed, showing great clinical application potential (Ceribelli et al., 2014). BRD4 has attracted attention as a promising anti-cancer drug target due to its strong effect on expression of the transcription factor MYC, which is a well-known pro-oncogenic master regulator and a major driver of wide variety of cancers (Cancer Genome Atlas Research Network, 2011; Cancer Genome Atlas Network, 2012a; Cancer Genome Atlas Network, 2012b; Cancer Genome Atlas Research Network, 2015). BET proteins (especially BRD4) have been shown to be effective in preclinical and clinical trials for lung cancer, breast cancer, leukemia, liver cancer, and multiple myeloma (Donati et al., 2018; Bauer et al., 2021). However, the study on the tolerance and toxicity of BET inhibitors is not comprehensive enough. Phase I drug has certain dose limiting toxicity (Bechter and Schöffski, 2020). The effectiveness of GBM treatment is limited by the blood brain barrier and resistance to single drug (Yang et al., 2021). Although the function of BET proteins have been investigated, the expression profile of BET proteins and the mechanism of BET proteins in GBM are still worth exploring. Furthermore, the unique functions of BET proteins and tumor immunology in GBM have not been fully elucidated.

In this article, first of all the disease-gene association, GO and KEGG pathways enrichment analysis of BET genes were evaluated in Metascape and David database, respectively. In addition, the expression level of BET genes in GBM tumor tissues and adjacent normal tissues was analyzed using the Oncomine and GEPIA database. Kaplan-Meier Plotter tool was used to evaluate the impact of BET mRNA expression on the prognosis of GBM patients. Moreover, TCGA and TIMER database were used to evaluate the immune checkpoints and immune infiltration levels of BET genes expression in GBM patients. ROC analysis of risk model was established to identify the correlation between BET genes and the prognosis in GBM patients. In conclusion, our study systematically described the expression profile of BET in GBM, and revealed the difference of BRD4 expression, which was over-expressed and significantly correlated with the prognosis of GBM patients. BRD4 may be a potentially valuable biomarker for prognosis evaluation of GBM.

## Materials and methods

### Metascape database analysis

Metascape (http://metascape.org) aims to provide a comprehensive gene list annotation and analysis resource for experimental biologists (Zhou et al., 2019). Metascape integrates multiple authoritative data resources such as GO, KEGG, UniProt and DrugBank, so that it can not only complete pathway enrichment and biological process annotation, but also perform gene-related protein network analysis and related drug analysis. In this study, Metascape was used for pathway analysis and prediction of adjacent genes related to BET proteins.

### David database analysis

DAVID 6.8 (https://david.ncifcrf.gov/home.jsp) is a bioinformatics database that integrates biological data and analytical tools to provide systematically integrated annotation information on biological functions for large-scale gene or protein lists. It can be used for differential analysis of genes and enrichment of pathways (Huang et al., 2009). In our study, GO and Reactome pathway enrichment analysis of BET proteins and related neighbor genes were screened out from DAVID database. The GO enrichment analysis included three parts: biological process (BP), cellular component (CC) and molecular function (MF). The Reactome pathway enrichment analysis was used to screen out potential molecular mechanisms.

### GEPIA database analysis

GEPIA, the gene expression profiling interactive analysis (http://gepia.cancer-pku.cn), is used to analyze RNA sequencing expression data of tumor and normal samples from TCGA and GTEx projects (Tang et al., 2017). GEPIA can perform single-gene analysis, cancer analysis, and multi-gene analysis. Single-gene analysis included whole body analysis, tumor stage analysis, survival analysis and co-expression analysis. The transcriptional level of BET proteins in GBM and normal tissue were calculated from GEPIA database. The correlation analysis of BRD4 and related genes in tissue on account of the TCGA and GTEx data. The overall survival (OS) or disease free survival (DFS) analysis of BET gene expression was counted by database. The multiple gene analysis can be compared in GEPIA 2 database.

### Oncomine database analysis

ONCOMINE (http://www. oncomine.org) is the largest oncogene microarray database and integrated data mining platform (Rhodes et al., 2004). The mRNA expression and DNA copy number of BET proteins family were analyzed by Oncomine database. The Oncomine platform contains 715 datasets and data from 86,733 cancer samples. The following conditions were used for analysis: Gene-BRD4; analysis type - cancer vs normal analysis; cancer type - Brain and CNS Cancer. In the study, two-fold change, *p*-value = 1E-4 and top 10% gene rank were selected as filter threshold. This analysis selected 11 datasets (2004 samples) which based on a series of Brain and CNS Cancer studies, including 62 datasets (5571 samples).

### The cancer genome atlas (TCGA) database analysis

TCGA database (https://portal.gdc.com) includes genome, transcriptome, epigenetic, proteome and other genomics data. The clinical information of GBM patients was downloaded from the database. The expression levels of eight genes were selected to be immune checkpoint, which were CTLA4,CD274,LAG3,SIGLEC15, HAVCR2, TIGIT, PDCD1 and PDCD1LG2. The transcription related to the immune checkpoint and the expression of these 8 genes were analyzed (Ravi et al., 2018; Wang et al., 2019). The above analysis methods and R package were implemented by R foundation.

### TIMER database analysis

TIMER database (http://cistrom.shinyapps.io/timer/) uses RNA-Seq expression profiling data to detect the infiltration of immune cells in tumor tissues. TIMER provides the infiltration of 6 types of immune cells (B-cell, CD4^+^ T-cell, CD8^+^ T-cell, Neutrphils, Macrophages and Myeloid dendritic cells) (Hoadley et al., 2014; Jin et al., 2021). The relations between BET genes and immune score was investigated with Spearman in TIMER database. The R software ggstatsplot package was used to plot the correlation between gene expression and immune score, and the R software heatmap package was used to plot the correlation between multiple genes. In this study, the “gene” module was used to evaluate the correlation between BET genes expression and immune cell infiltration, and the “SCNA” module was used to compare the level of immune infiltration in GBM with different changes in somatic cell copy numbers.

### Correlation of BRD4 and immune infiltration level

An integrated repository portal for tumor-immune system interactions (TISIDB, http://cis.hku.hk/TISID B/index. php) was used to examine tumor and immune system interactions in 28 types of tumor-infiltrating lymphocytes (TILs) across human cancers. The relative abundance of TILs were inferred by using gene set variation analysis (GSVA) based on BRD4 expression.

### The receiver operating characteristic analysis

The clinical information of Glioma samples was obtained from TCGA and ICGC database. The least absolute shrinkage and selection operator (LASSO) regression algorithm was used for feature selection, 10-fold cross validation and R software package were used for the above analysis (Zhang et al., 2020). The patients were divided into high-risk group and low-risk group with the median risk score as the critical value. The ROC curve was generated to predict the accuracy of patients’ OS through time-dependent ROC analysis. In the KM curves, hazard ratio (HR) with 95% confidence interval (CI) and *p*-values were generated by logrank tests.

The prognostic of BRD4 gene on a single sample (Pediatric Brain Tumor- Multiple subtypes, 251 cases) was studied in the ICGC training set, the samples was ranked from high to low expression of this gene, and the prognosis differences of different groups was analyzed. The worse the prognosis of high expression, the better the gene may promote tumor development.

The expression levels and regression coefficients of the five genes were combined using the linear combination method to obtain the risk score formula, as follows: Risk score = $\sum {}_{i=1}^{5}\beta i*Expi$ where Exp was the expression level of each prognostic gene, and β was the regression coefficient.

### Development of a nomogram and clinical characteristics analysis

The corresponding clinical information for BET genes were downloaded from the TCGA dataset (https://portal.gdc.com). The nomogram was used to study the influence of genes, clinical factors (such as age, sex, stage, *etc.*) on the prognosis. First of all, if there was significance in single factor cox regression analysis, which was considered that this variable was related to the prognosis; At the same time, if this variable was also significant in multivariate cox regression analysis, it can be considered as an independent prognostic factor. Univariate and multivariate cox regression analysis were used to identify the proper terms to build the nomogram. The nomogram according to BET gene and clinical characteristics was performed by “rms” package. The forest was used to show the *p*-value, HR and 95% CI of each variable by “forestplot” R package. The BET expression and the clinical characteristics of GBM were compared with survival status and survival time to determine whether the BET gene expression can be regarded as an independent prognostic factor.

### Immunohistochemistry analysis

The Human Protein Atlas (HPA) (https://www.proteinatlas.org) aims to provide 17,268 unique proteins distribution information in different tissues and cells. The pathology part contains the pathological information of mRNA and protein expression data of human cancer, as well as millions of immunohistochemical staining results (Sjostedt et al., 2020). In the study, in order to compare the expression of BET proteins in normal tissues and tumor tissues, immunohistochemical staining images of genes were collected from HPA database. ImageJ software was used for immunohistochemical quantitative analysism, the area, mean density and integrated optical density (IOD) of positive cells was calculated in each group.

### LinkedOmics database analysis

LinkedOmics database (http://www.linkedomics.org/login. php) includes multi-omics data and clinical data for 32 cancer types. The database can not only analyze gene expression, but also analyze gene and tumor patients’ prognosis and survival, protein phosphorylation, gene methylation, non-coding RNA related genes and other data. KEGG and Gene Set Enrichment Analysis (GSEA) can also be analyzed (Vasaikar et al., 2018). The LinkFinder module was used to analyze the differential expression of BRD4 related genes in the TCGA GBM cohort (*n* = 528). GSEA was used to analyze the GO and KEGG pathways.

### Immunofluorescence analysis

The principle of immunofluorescence experiment is to label fluorescein on antibody and directly react with the corresponding antigen. The expression of BRD4 in neuroglial cells and U-251 MG cells was analyzed by immunofluorescence staining. The logarithmic growth cells were taken, washed twice with PBS (1000rpm, 5 min) by centrifugation, and the cell sheets were prepared by a cytocentrifugation machine or directly prepared cell smears. Then cells were incubated with an anti-BRD4 (1:200, ab128874, Abcam, Tianjin, China) antibody overnight. Then, the diluted fluorescent secondary antibody (1:1000, ab97023, Abcam, Tianjin, China)was added dropwise and incubated for 1 h. DAPI was added dropwise and incubated in the dark for 5 min, the specimens were stained, and the slides were sealed with a mounting liquid containing an anti-fluorescence quencher, and then the images were collected under a fluorescence microscope (Olympus BX53, Japan). The expression of BRD4 was semi quantitative using the specific fluorescence intensity. The average fluorescence intensity of fluorescence photos taken by laser confocal microscope was detected by ImageJ software. Mean gray value (Mean) = Integrated density (IntDen)/Area.

### Western blot analysis

Cells were collected and lysed to extract total protein. After quantification, the same amount of protein was separated by SDS-PAGE electrophoresis, and then the protein was transferred to polyvinylidene fluoride membrane (PVDF). The membranes were blocked in blocking solution for 2 h and incubated with BRD4 (1:1000, ab128874, Abcam, Tianjin, China) primary antibody at 4°Covernight, PBS washed the membranes. Then added secondary antibody (1:5000, ab97023, Abcam, Tianjin, China)and incubated for 2 h at room temperature, developed with ECL luminous color developer. β-Actin was used as internal reference. Gel-pro2020D-I analysis software was used to digitize the gray value of each special band on the picture. The gray value of the target protein/the internal Actin to correct the error, and the result represents the relative content of the target protein. Each assay was performed in triplicate.

### Statistical methods

Chi-square test was used to evaluate the correlation between molecular subtypes and routine clinical variables. Benjamin-Hochberg’s FDR was used to correct multiple tests. The log-rank tests was used to evaluate survival scores. These statistics are carried out using R software.

## Results

### Enrichment analysis of BET proteins functional networks in GBM

**FIGURE 1:**
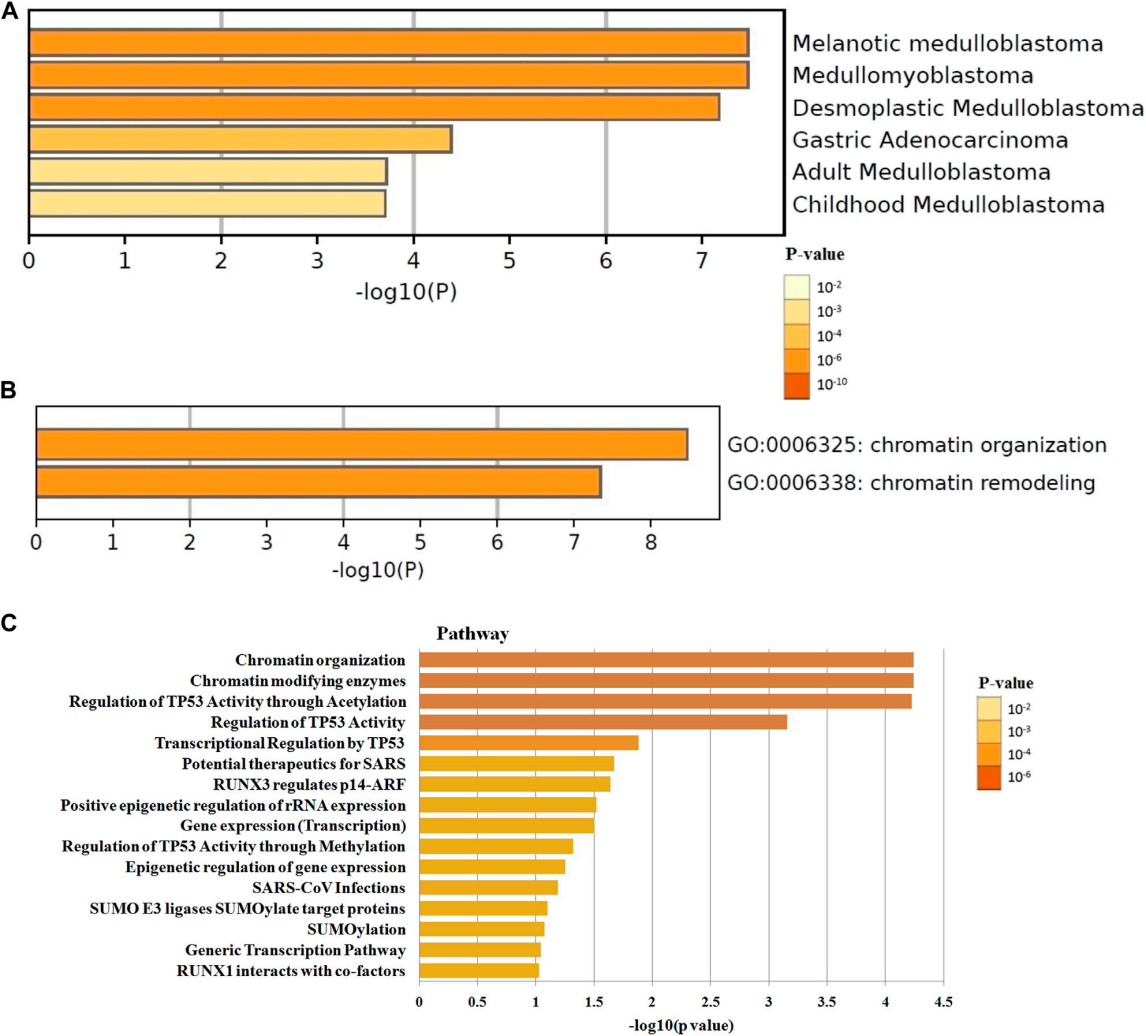
The enrichment analysis of BET genes were predicted by bioinformatics analysis. **(A)** The top few enriched clusters were identified in DisGeNET ontology categories in Metascape database. **(B)** The Go enrichment heatmap was plotted with *p*-value in Metascape database. **(C)** The Reactome pathway enrichment analysis of BET co-expression genes in David database. The more abundant the gene, the darker the color.

The differential expression function and pathway enrichment of BET genes in GBM were analyzed by Metascape and GEPIA databases. Gene list enrichment was identified in the ontology category of DisGeNET. The results indicated that human disease-associated genes might be closely related to medulloblastoma and adenocarcinoma **(** Figure 1A). Medulloblastoma was a type of central nervous system tumor, which showed a strong association between BET genes and brain tumors. The top 2 GO enrichment items of BET genes focused on biological processes were chromatin organization and chromatin remodeling**(** Figure 1B). The top 10 related genes of BET were identified through GEPIA dataset (Table 1).

**TABLE 1:** The top 10 significant genes correlated with differentially expressed BET genes in GBM (GEPIA).

Gene	Correlated genes
BRD1	TTC28, ZBED4, PPP6R2, TCF20, PRR14L, MORC2, TNRC6B, NUP50, ZNF70, GTPBP1
BRD2	TRIM26,DHX16,ABCF1,SAFB,RBM10,LEMD2,COPS7B,TRIM39,UBR2,DVL3
BRD3	GTF3C4,CAMSAP1,EHMT1,PRRC2B,PRDM10,GATAD2B,FAM168B,PHF2,RC3H2,PBRM1
BRD4	WIZ,AP3D1,ARHGEF18,NACC1,KHSRP, GATAD2A,CHERP, TNPO2,SAFB,MAU2
BRDT	NUPL1P1,CRYGC,CTA-150C2.13,RP11-1281K21.8,RP5-1185K9.1,CTC-420A11.2,AMELX,RP11-532F6.2,RNA5SP218,RP11-1267H10.1

The most highly enriched Reactome pathways were calculated by DAVID 6.8 (Figure 1C). As expected, among the top 10 Reactome pathway: chromatin organization, chromatin modifying enzymes, regulation of TP53 activity, transcriptional regulation by TP53, potential therapeutics for SARS, RUNX3 regulates p14-ARF, positive epigenetic regulation of rRNA expression, gene expression, epigenetic regulation of gene expression, and SUMO E3 ligases SUMOylate target proteins were significantly associated with the tumorigenesis. The pathway of potential therapeutics for SARS, RUNX3 regulates p14-ARF, and SUMO E3 ligases may be involved in the regulation of immune system.

### Aberrant expression of BET genes in GBM

**FIGURE 2:**
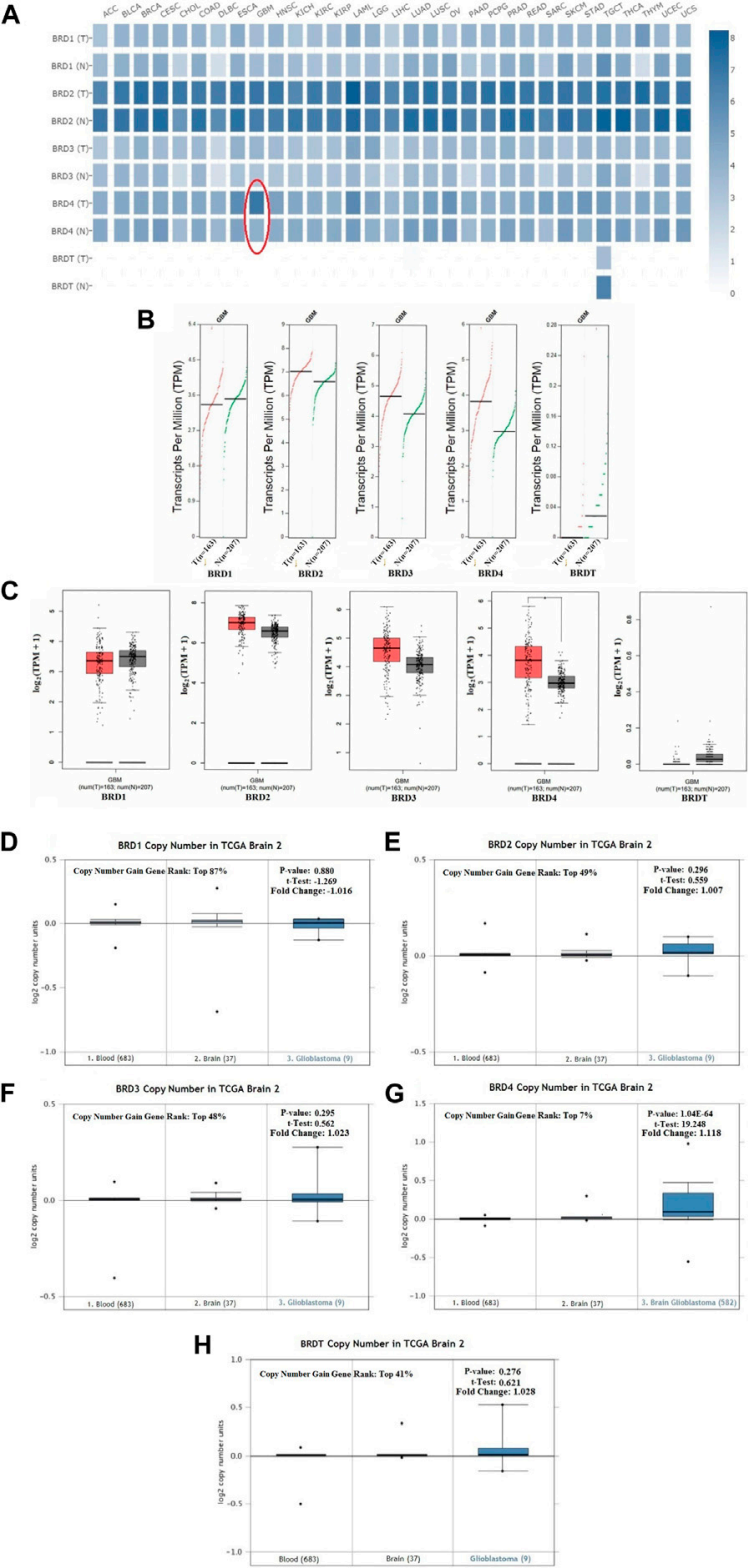
The transcription levels of BET genes in GBM samples. **(A)** Multiple gene comparison in different cancer types were generated in GEPIA database. T:Tumor tissues; N: Normal tissues. **(B)** The transcription level of BET genes in GBM patients. The scatter diagram of expression of BET genes in GBM. **(C)** The box plot of BET genes expression in GEPIA database (*p < 0.05). **(D–H)** The copy number of BRD1, BRD2, BRD3, BRD4, BRDT were shown in TCGA Brain 2 cohort, respectively. The *p*-values, *t*-Test, fold change, and copy number gain gene rank were based on Oncomine database.

The GEPIA dataset was used to compare the BET mRNA expression between tumor and normal tissues. The gene list was inputed in the list, all cancer species were selected, and generated the polygene analysis map. The transcriptional levels of BET (BRD1,BRD2,BRD3, BRD4 and BRDT) were compared between cancer and normal samples in GEPIA database. This feature provided expression matrix plots based on BET genes. The mRNA expression levels of BRD4 was more significantly increased in GBM patients than other types of cancer (Figure 2A **)**.

Comparison of BET mRNA expression between GBM tumor and normal tissues in GEPIA database. The results indicated that the expression levels of BRD2, BRD3, BRD4 were higher in GBM tumor tissues than those in normal tissues, and BRDT expression in tumor was lower than that in normal tissues. The expression of BRD4 was more significantly increased in GBM cancer tissues than other gene **(** Figures 2B,C **)**.

This study preliminarily evaluated the transcriptional level of BET genes in several brain studies of TCGA. The Oncomine data showed that DNA copy number variation (CNV) of BRD4 in GBM tissues was significantly higher than that in normal brain tissue and blood (*p* ˂ 0.01). The copy number gain gene rank of BRD1,BRD2, BRD3, BRD4 and BRDT was 16,261(in top 87%),9063(in top 49%),9028(in top 48%), 1306(in top 7%) and 7634 (in top 41%), respectively (Figures 2D–H). The over-expression gene rank of BRD1,BRD2, BRD3, BRD4 and BRDT was 6451(in top 52%),4946(in top 40%),5998(in top 48%), 3054(in top 16%) and6196 (in top 50%), respectively (Supplementary Figures S1A–E). Despite fold difference within 2, the gene rank of BRD4 was still in the top 7% based on DNA CNV and in the top 16% based on expression, which was better than that of other BET genes in GBM. Therefore, the expression of BRD4 can be used as a potential diagnostic indicator in GBM.

### The prognostic value of BET genes in GBM

**FIGURE 3:**
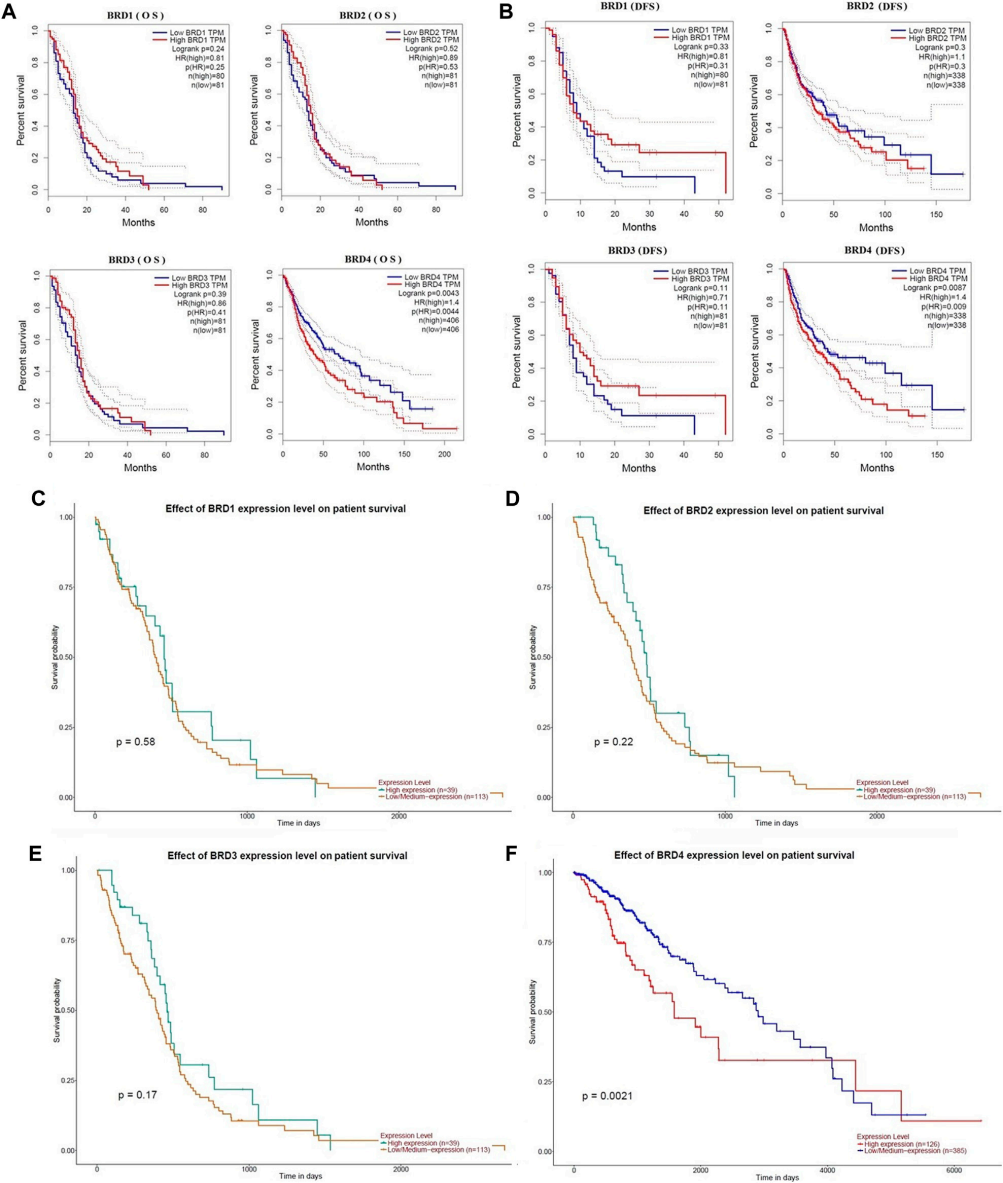
The prognostic value of BET genes in GBM patients from the GEPIA database. The survival curves were plotted using the Kaplan–Meier Plotter. **(A)** The overall survival rate (OS) of BRD1, BRD2, BRD3 and BRD4 mRNA, respectively. **(B)**The disease-free survival rate (DFS of BRD1, BRD2, BRD3 and BRD4 mRNA, respectively. **(C–F)** The effect of BRD1, BRD2, BRD3 and BRD4 expression level on patient survival from UALCAN database, respectively.

The critical efficiency of BET genes were explored in the survival of GBM patients in GEPIA database. The correlation between BET mRNA levels and GBM patient survival was analyzed using the Kaplan-Meier plotting tool. The survival curves for high expression (red) and low expression (blue) of BET genes were plotted in GBM patients. The Kaplan-Meier curve analyses showed that decreased BRD4 mRNA levels were significantly associated with overall survival (OS) and disease-free survival (DFS) in GBM patients (Figure 3; *p* < 0.01). The GBM patients with low expression of BRD4 predicted better OS and DFS. Therefore, survival analysis showed that decreased BRD4 expression was significantly associated with improved prognosis in GBM patients.

### Distinct characteristics of immunogenicity of BET genes in GBM

**FIGURE 4:**
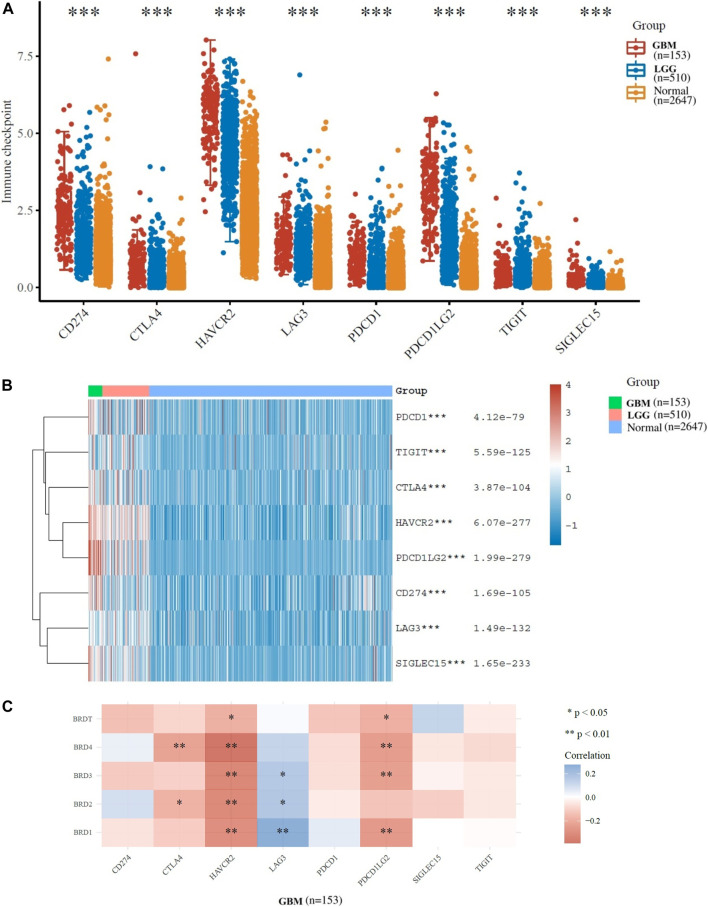
Immunological checkpoint related transcriptional level and expression in Glioma samples in TCGA dataset. **(A)**The expression distribution of immune checkpoints gene (CD274, CTLA4, HAVCR2, LAG3, SIGLEC15, TIGIT, PDCD1 and PDCD1LG2) in tumor and normal tissues. **(B)**The heatmap of gene expression related to immune checkpoint (*p < 0.05, **p < 0.01, ***p < 0.001). **(C)**The correlation between BET genes expression and immune checkpoint gene expression in GBM.

Options for partial correlation conditioned on tumor purity was provided. The expression profiles of 8 immune checkpoint genes critical for immune regulation (CD274, CTLA4, HAVCR2, LAG3, SIGLEC15, TIGIT, PDCD1 and PDCD1LG2) were further examined. The expression of eight genes were significantly higher in GBM and LGG patients compared to normal patients (Figures 4A,B). The results indicated that the expression of BET gene was significantly correlated with immune checkpoint of 153 patients with GBM (Figure 4C). The expression of BRD1, BRD2 and BRD3 were significantly positively correlated with LAG3 (Cor = 0.275, *p* = 5.98e−04), (Cor = 0.172, *p* = 3.38e−02) and (Cor = 0.164, *p* = 4.29e−02), respectively. The expression of BRD2 and BRD3 was significantly negatively correlated with HAVCR2 (Cor = −0.309, *p* = 1.06e−04) and (Cor = −0.319, *p* = 6.33e−05), respectively. The expression of BRD4 was significant negatively correlated with CTLA4 (Cor = -0.227,*p* = 4.75e−03) and HAVCR2 (Cor = −0.391, *p* = 7.62e−07) and PDCD1LG2 (Cor = -0.244,*p* = 2.48e−03). Therefore, The expression of BET genes closely related to the immune checkpoint genes in GBM.

**FIGURE 5:**
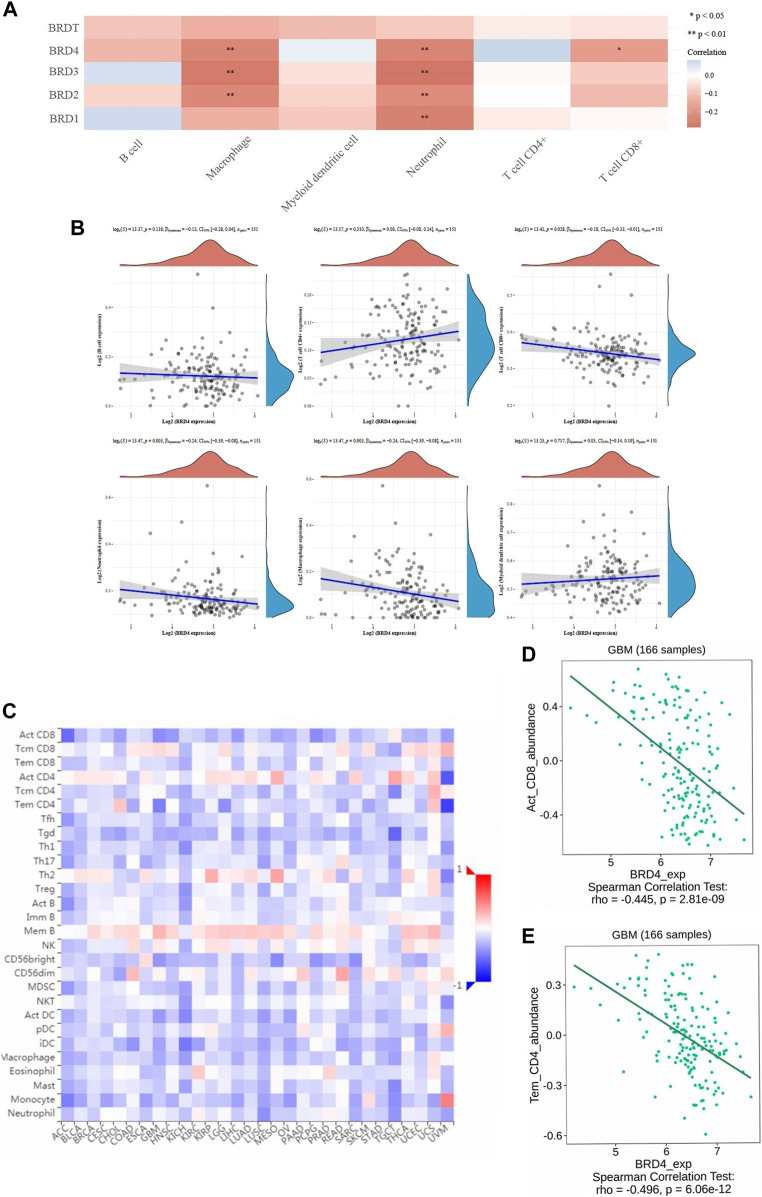
The correlation between BET genes expression and immune score was analyzed in TIMER and TISIDB database. **(A)** The heatmap of the correlation between BET genes and immune score in six types of immune cells. The different colors indicated different correlation coefficients (blue for positive correlation and red for negative correlation). **(B)** Relationships between the immune infiltration abundances and BRD4 expression in different immune cells (B-cell, CD4+T-cell, CD8+T-cell, neutrophils, macrophages and myeloid dendritic cells). **(C)** The correlations between expression of BRD4 and 28 types of TILs across human heterogeneous cancers. **(D)** BRD4 significantly negative correlated with abundance of activated CD8 T-cell (Act_CD8; rho = −0.445, *p* < 0.001). **(E)** BRD4 significantly negative correlated with abundance of effector memeory CD4 T-cell (Tem_CD4; rho = −0.496, *p* < 0.001).

TIMER database was used to analyze tumor infiltrating immune cells (including B-cell, CD4+T-cell, CD8+T-cell, macrophages, neutrophils and myeloid dendritic cells). The immune cell analysis was performed to determine changes in the tumor microenvironment resulting from differential expression of BET proteins (Figure 5A). In the study of six types of immune cells, BRD4 gene was significantly higher correlation effect than other BET genes, which represented negative correlation. The macrophage (log_e_(*S*) = 13.47, *p* = 0.003, CI_95%_ [−0.39, −0.08]), neutrophil (log_e_(*S*) = 13.47, *p* = 0.003, CI_95%_ [−0.39, −0.08]), and CD8^+^ T-cell expression (log_e_(*S*) = 13.42, *p* = 0.028, CI_95%_ [−0.33, −0.01]) showed the significant difference in infiltration abundance with BRD4 expression in GBM patients (Figure 5B, *p* < 0.05). The level of BRD4 expression in BET was significant correlation with the level of immune infiltration. The expression of BRD4 was positively correlated with tumor purity, and negatively correlated with immune infiltration abundance of macrophage, neutrophil and CD8 + T-cell, respectively. Additionally, The significant correlation of with 28 types of TILs was performed across human cancers (Figure 5C). BRD4 was significantly correlated with abundance of activated CD8 T-cell (Act_CD8; rho = −0.445, *p* < 0.001), and effector memeory CD4 T-cell (Tem_CD4; rho = −0.496, *p* < 0.001) of TILs across human heterogeneous cancers (Figures 5D,E). The results showed that BRD4 gene was negatively correlated with most TILs.Therefore, the BRD4 was significantly negatively correlated with immune infiltration in BRD family genes.

**FIGURE 6:**
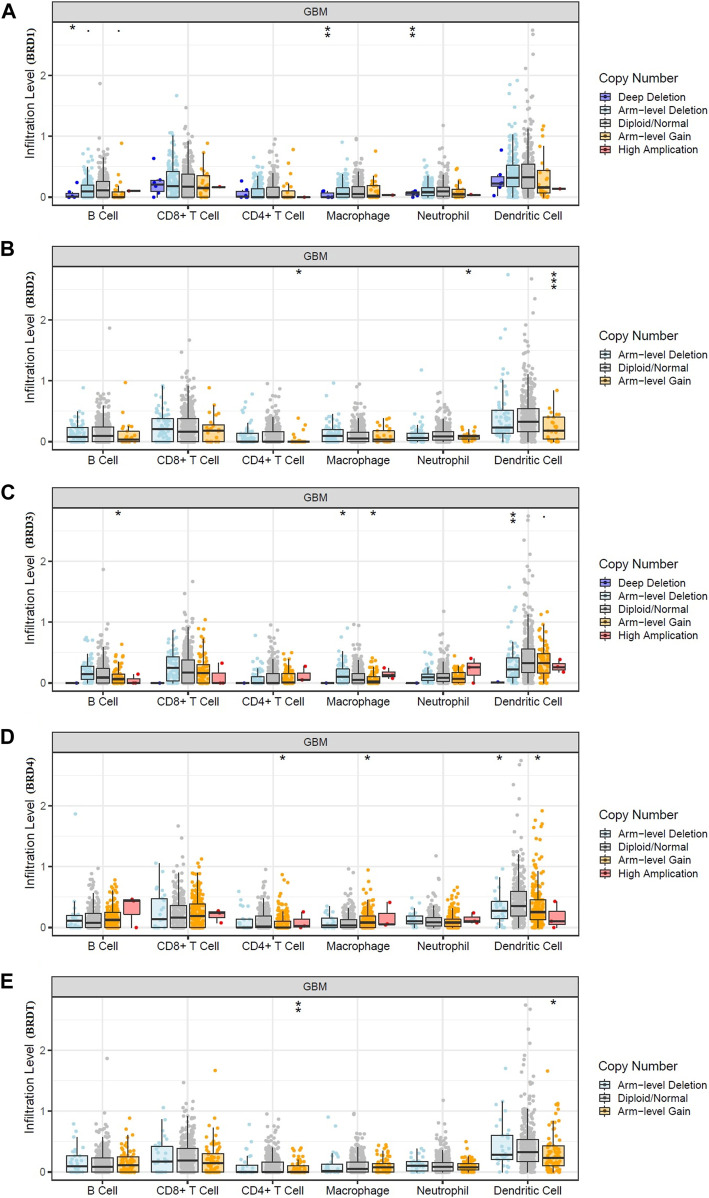
Effect of gene copy number changes expressed by different BET genes expression on immune cell infiltration in TIMER database. **(A)** BRD1, **(B)** BRD2, **(C)** BRD3,**(D)** BRD4,**(E)** BRDT.

The bilateral Wilcoxon rank-sum test was used to compare the infiltration level of each SCNA category with the normal value. The SCAN module was used to study immune cell infiltration caused by gene copy number alterations in different BET genes expression (Figure 6). Our results suggested that alterations in gene copy number could affect immune cell infiltration. Overall, the expression of BRD4 in BET genes may play a key role in tumor development by participating in immune response.

### Prognostic analysis of risk score in GBM

**FIGURE 7:**
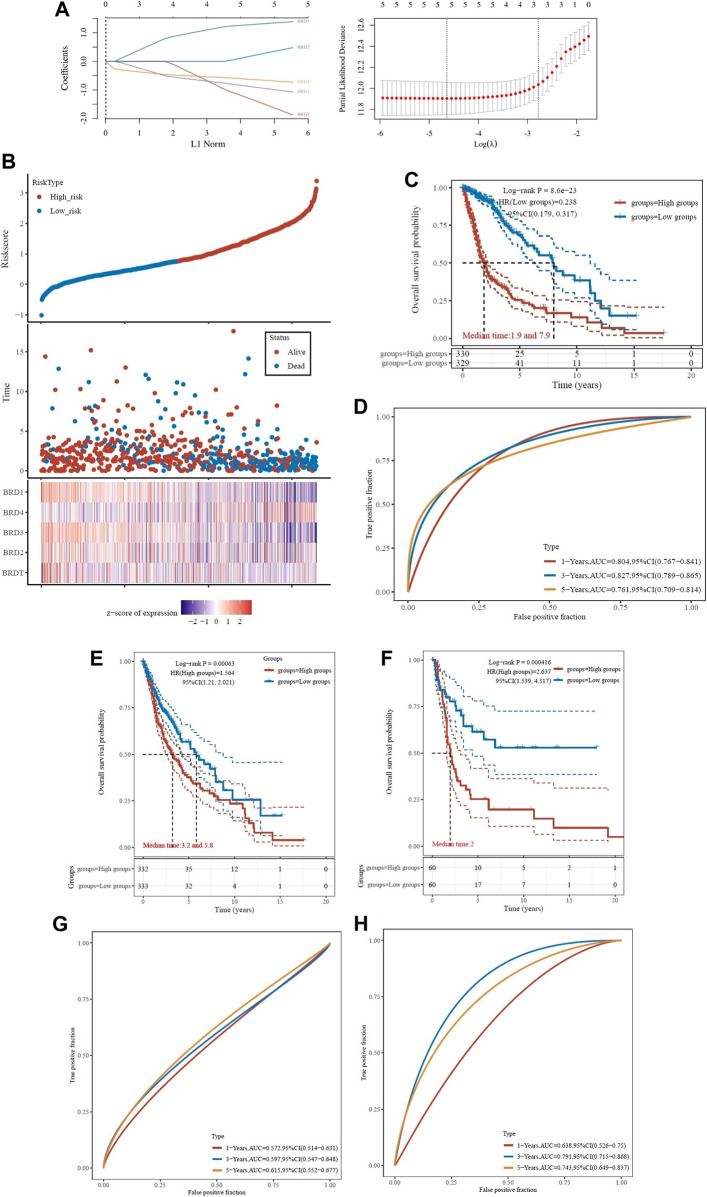
Analysis of prognostic assessment of BET genes in TCGA dataset **(A–D)**, and external validation of BRD4 gene signature in ICGC training set **(E–H)**. Patients were divided into low-risk and high-risk groups. **(A)** LASSO regression analysis was performed on BET genes to calculate the correlation coefficients. Coefficients of selected features are shown by lambda parameter; Partial likelihood deviance *versus* log(λ)was drawn using LASSO Cox regression model. **(B)** The LASSO algorithm was used to generate risk scores for the training cohort from TCGA. Relationship of BET proteins expression with risk score, survival time, and survival status were shown in the training cohort. **(C)** Distribution of KM survival curves for differential expression of BET proteins in the training cohort. **(D)** ROC curve and AUC of BET proteins signature classification. **(E)** Kaplan-Meier survival analysis of patients in different risk groups from TCGA dataset. **(F)** Kaplan-Meier survival analysis of patients in different risk groups from ICGC training set. **(G)** The ROC curves for risk score in TCGA dataset. **(H)** The ROC curves for risk score in ICGC training set. The higher values of AUC corresponding to higher predictive power.

To determine the association of BET genes with the prognosis of GBM patients, the ROC risk analysis model was established. Ten fold cross validation of LASSO regression was performed to obtain the best lambda value (λmin = 0.0102), which was related to 5 genes significantly associated with OS in DEGs (Figure 7A). Therefore, the risk score for five genes was constructed according to the Cox coefficient: risk score=(−1.0055)* Exp (BRD1)+ 0.399* Exp (BRD2)+(−0.6401)* Exp (BRD3) +1.3192* Exp (BRD4) +(−1.5436)* Exp (BRDT). Median cutoff points were obtained using the R package and patients were divided into high-risk group (*n* = 330) and low-risk group (*n* = 329). The heatmap showed the correlation between the survival status of all patients in the sequence and the five prognostic genes (Figure 7B).

The KM survival curve showed that the OS of high-risk group was worse than that of low-risk group, which indicated that the low-risk group had significant difference in logrank *p* < 0.01, HR = 0.238 (0.179–0.317). The OS of the samples with higher risk score was significantly smaller than that of the lower risk score, indicated that the higher the risk score, the worse the prognosis (Figure 7C). The ROC of risk score was analyzed using the R software package timeROC, and the classification efficiency of 1-year, 3-year and 5-year prognosis was analyzed (Figure 7D **)**. The AUC of this model was greater than 0.7, indicated that the polygenic model had better predictive power in 1, 3, and 5-year OS. Univariate Cox analysis showed that the model had good survival prediction and prognosis discrimination ability.

Due to the external validation of BRD4 gene signature in ICGC training set, the risk score was calculated for each patient, and patients in the two validation sets were divided into high- and low-risk groups based on the median risk score. The results showed that the survival rate was lower in the high-risk group (Figures 7E,F). The AUC values of the ROC curves for the 1-, 3- and 5-year survival rates were 0.572, 0.597 and 0.615 in TCGA dataset (Figure 7G), and 0.638, 0.791, and 0.743 in ICGC training set (Figure 7H), respectively. Based on the risk score, the prognostic model had high accuracy and stability in brain cancer disease prognosis assessment.

ROC curve was used to evaluate the predictive effect of each BRD family member on GBM prognosis. The KM survival curve showed that the OS of BRD4 with high-risk group was worse than that of low-risk group. The AUC values of BRD4 ROC curves for the 1-, 3- and 5-year survival rates were 0.604, 0.725, and 0.636, respectively. The results also showed that BRD4 had significant difference (logrank *p* < 0.05) in the prognosis of GBM, while BRD1, BRD2, BRD3 and BRDT had no significant difference (Supplementary Figure S2).

### Establishment and validation of BET nomogram

The nomogram was constructed by BET genes and the clinicopathological characteristics in the cohort of TCGA-GBM. The results showed that the distribution of different clinical indicator and BRD4 expression in all samples had different contributions in the whole scoring process (Supplementary Figure S3A). In addition, the OS of 2 and 3 years was predicted and analyzed, which showed that the nomogram had good prediction ability (Supplementary Figure S3B). The univariate (Supplementary Figure S3C) and multivariate (Supplementary Figure S3D) Cox regression analysis showed that BRD4 expression, age and grade had a significant correlation with OS. The univariate analysis and multivariate analysis showed that BRD4 was an independent prognostic factor for TCGA-GBM patients.

### The immunohistochemistry analysis of BET proteins

**FIGURE 8:**
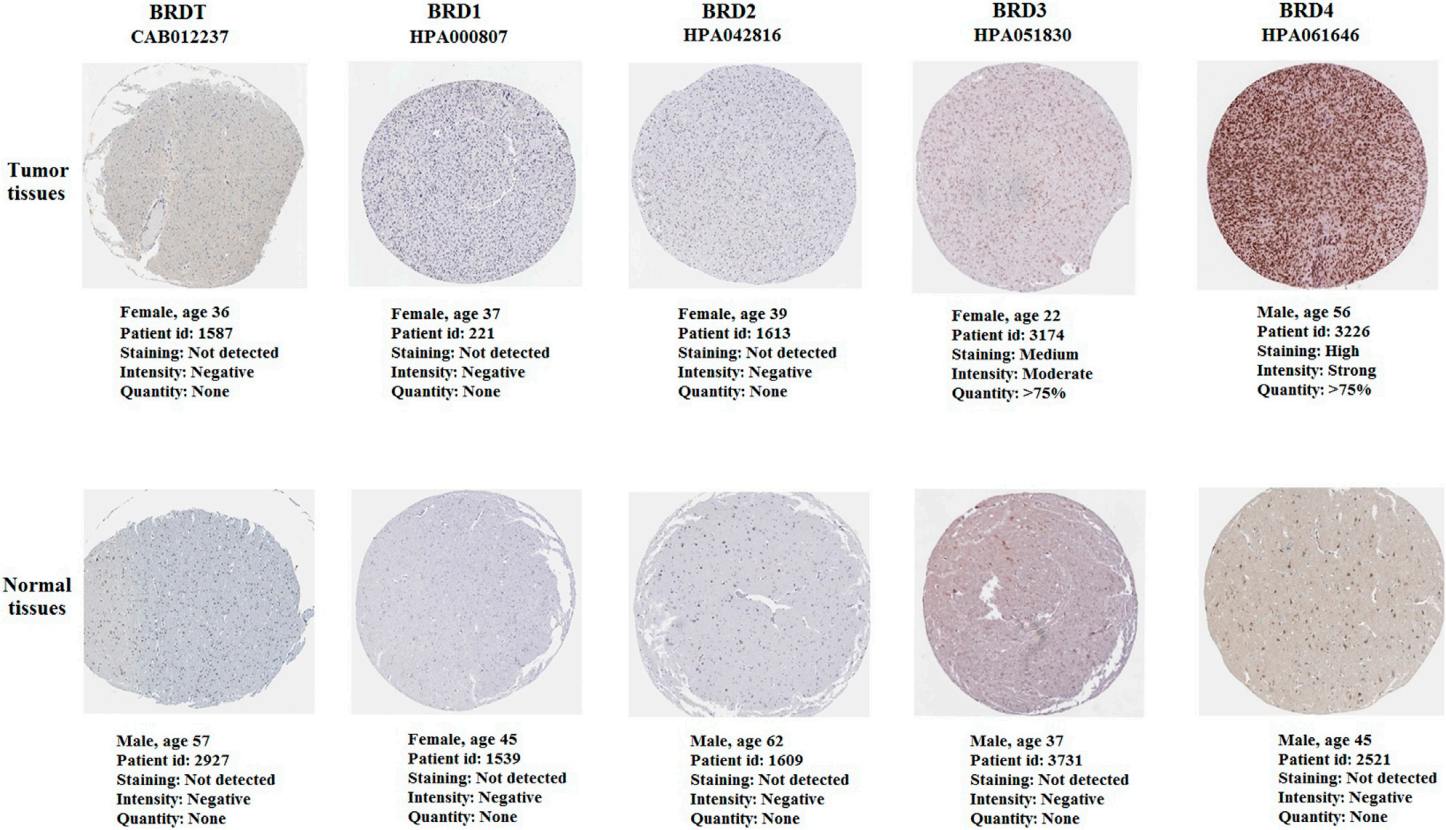
The immunohistochemistry of BET proteins in tumor tissues and normal cerebral cortex tissues (Scale bar: 200 μm). The expression of BRD4 protein in tumor tissues was higher than that in normal tissues.

The immunohistochemistry (IHC) results of the HPA database were used verify the difference in BET protein expression. By comparing the density, intensity and total quantity of staining, it was showed that BRD3 and BRD4 expression levels in GBM tissues were higher than those in normal cerebral cortex tissues (Figure 8). The study found that BRD4 protein was significantly higher expressed in tumor tissues than in normal tissues. There was no significant difference in the expression of BRDT, BRD1 and BRD2 in tumor tissue and normal tissue.

### Enrichment analysis of BRD4 gene functional networks in GBM

**FIGURE 9:**
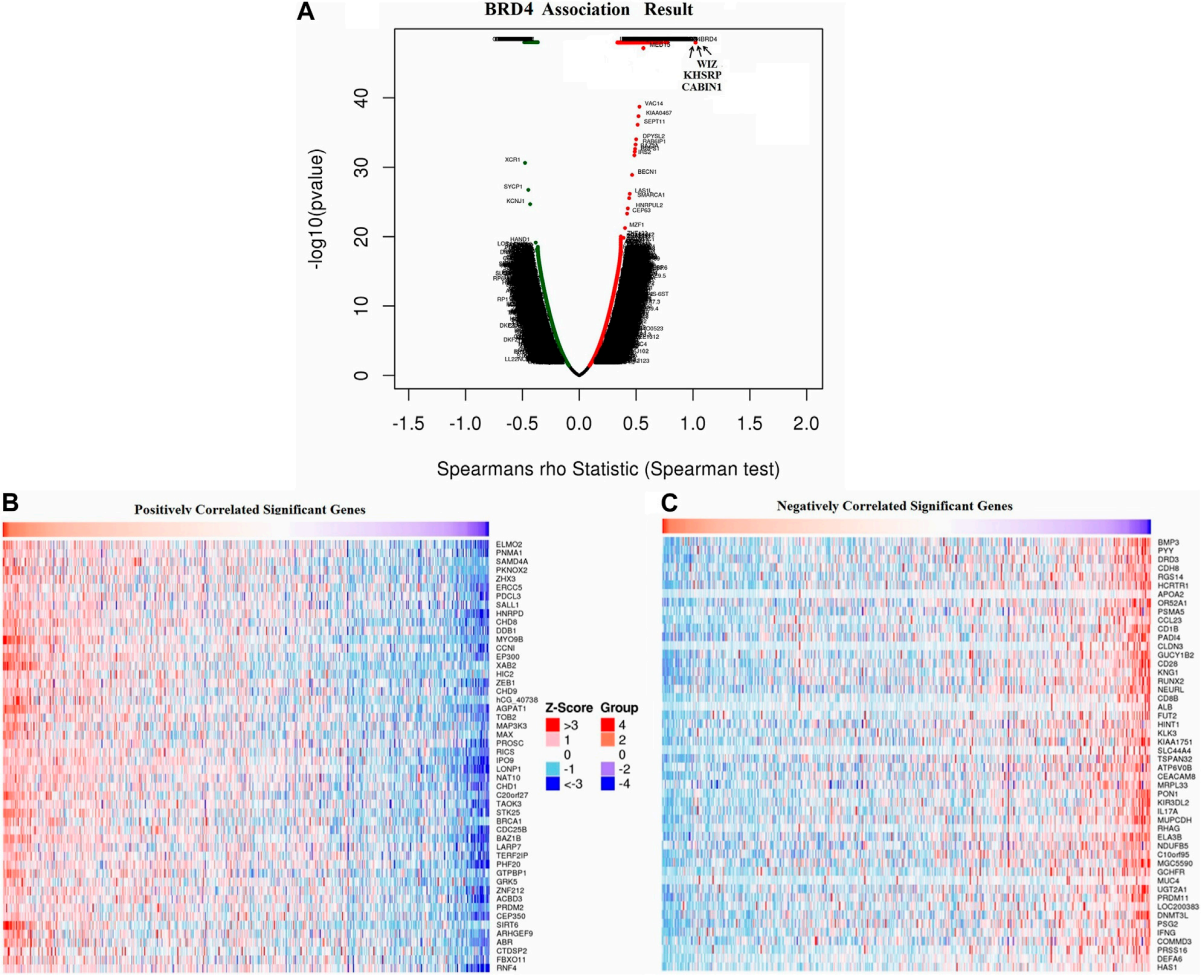
Differentially expressed genes associated with BRD4 in GBM samples in LinkedOmics database. **(A)** The correlation between BRD4 and differentially expressed genes in GBM was analyzed by Spearman test. **(B–C)** Heatmaps showed the positive and negative correlation genes (top 50) with BRD4 in GBM.

Overall, the expression of BRD4, one of the BRD family genes, may play an important role in tumor development. So we focused on the mechanism and signal pathway of BRD4 gene. Function module was used to analyze mRNA sequencing data of 528 GBM patients in TCGA. The volcano plot showed that 8780 genes were significantly positively and negatively correlated with BET genes (Figure 9A, false discovery rate [FDR] < 0.01). Heatmap showed that 50 significant genes were positively and negatively correlated with BRD4 expression (Figures 9B,C). The results showed a strong positive correlation between the expression of BRD4 and WIZ (Spearman correlation = 0.7719, *p* = 1.000e-48), KHSRP (Spearman correlation = 0.7555, *p* = 1.000e-48), CABIN1 (Spearman correlation = 0.7244, *p* = 1.000e-48), and GNA11(Spearman correlation = 0.7237, *p* = 1.000e-48), which reflected changes in regulation of RNA metabolic process, activating transcription factor, DNA Damage and RNA degradation.

**FIGURE 10:**
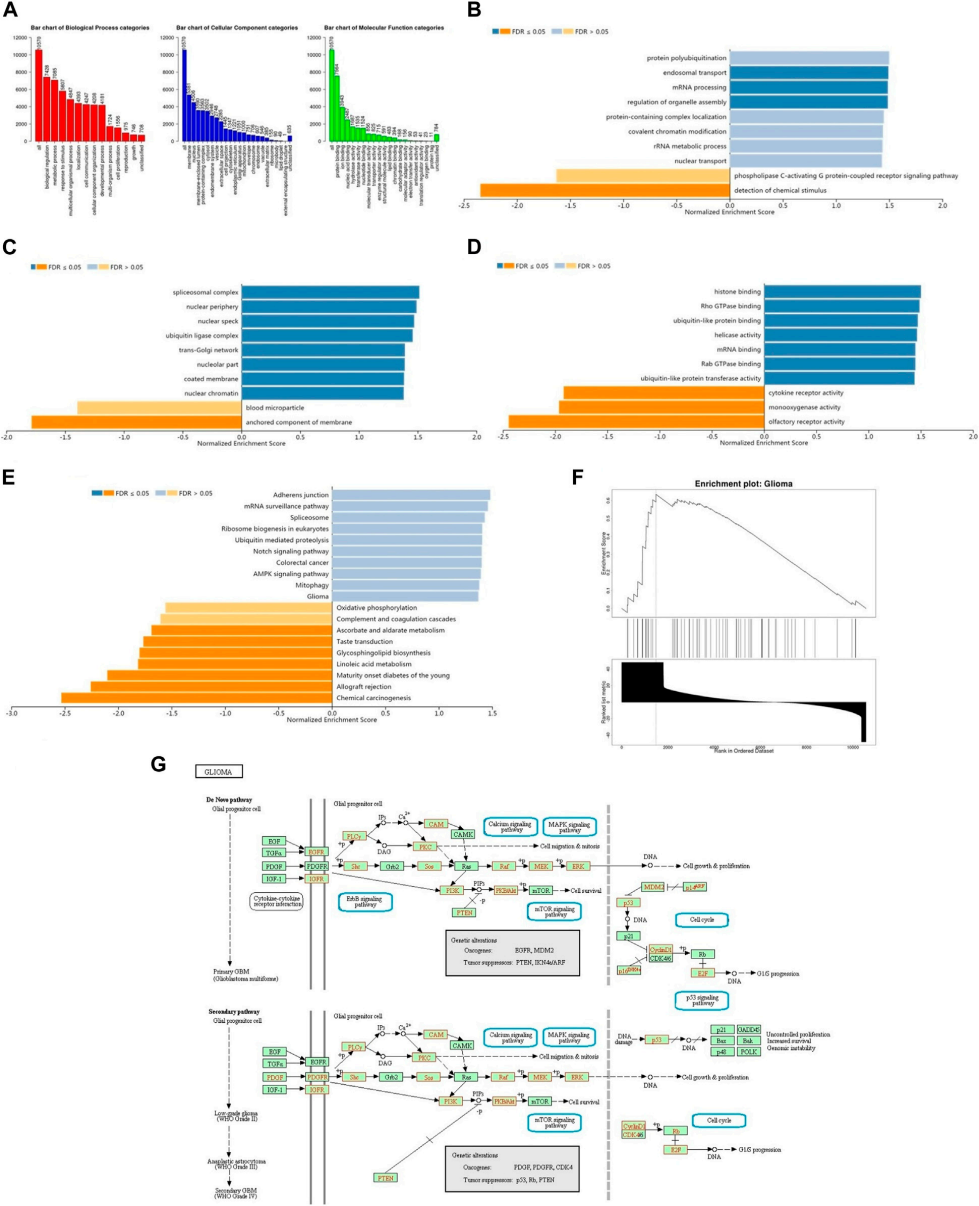
The function of BRD4 gene was predicted by the analysis of GO and KEGG in GSEA. **(A)** The GO enrichment analysis of target genes was predicted from three aspects: biological process (BP), cellular component (CC) and molecular function (MF). **(B)** The top 10 functional roles of BP for BRD4. **(C)** The top 10 functional roles of CC for BRD4. **(D)** The top 10 functional roles of MF for BRD4. **(E)** KEGG analysis of differentially expressed genes. **(F)** Enrichment analysis of KEGG pathway showed the regulation pathway of Glioma. **(G)** KEGG annotations of the Glioma pathway regulated by BRD4 in brain cancer (cBioPortal). Targets marked in red were related to the Leading Edge Gene.

The enrichment analysis of GO in GSEA showed that the differentially expressed genes related to BRD4 were mainly located in biological regulation, protein/nucleic acid binding and cell communication, which acted as transcription factor and molecular transducer (Figures 10A–D). KEGG pathway analysis showed enrichment in 19 most significant categories and representatives in the reduced sets. It was closely related to mRNA surveillance pathway, ubiquitin mediated proteolysis, notch signaling pathway, colorectal cancer, AMPK signaling pathway, mitophagy, oxidative phosphorylation, glioma pathway and so on (Figure 10E). There were 18 leading Edge Num in the Glioma pathway, such as AKT1, ARAF, CALM1,CCND1,E2F3,IGF1R, MAP2K2, MAPK1, PIK3CA,PIK3R1,PIK3R2,PLCG1,PRKCA,RAF1,SHC2,SHC3, SOS2 and TP53 (Figure 10F). Specific signal pathways were involved in the Glioma, such as Cell cycle, MAPK signaling pathway, Calcium signaling pathway, p53 signaling pathway, mTOR signaling pathway and so on (Figure 10G).

### BRD4 expression is significantly increased at the cellular level in GBM

Neuroglial cells and U-251 MG cells were obtained from the Institute of Radiation Medicine (Tianjin, China), which were cultured in RPMI 1640 medium and 10% fetal bovine serum (Bioroc Pharmaceutical & Biotech, Tianjin, China).

**FIGURE 11:**
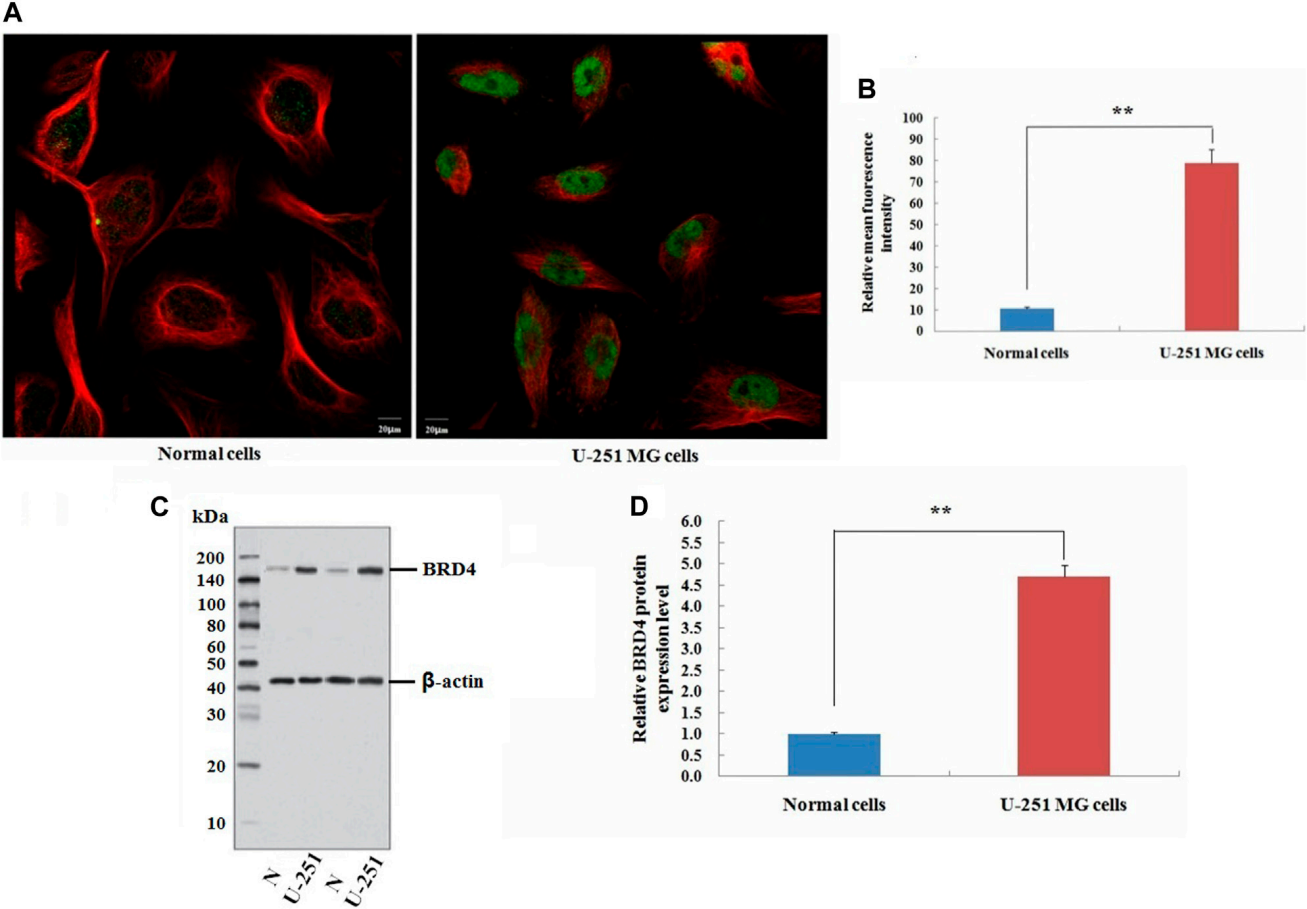
Validation experiment of BRD4 expression in cells. **(A)** Immunofluorescence analysis of BRD4 (green) in normal neuroglial cells and U-251 MG cells (Scale bar: 20 μm). **(B)** Mean relative fluorescence intensity values for each group. **(C)** Western blot analysis of BRD4 protein between normal neuroglial cells and U-251 MG cells. β-Actin was an internal control. **(D)** Relative expression value of BRD4 protein (means ± SD, *n* = 3,**p < 0.01).

This study also carried out immunofluorescence experiments to verify that BRD4 showed green fluorescence in tumor cells, indicating the high expression (Figures 11A,B). Next, the expression level of BRD4 in U-251 MG cells and normal neuroglial cells was detected by western blots. Consistent with the analysis of TCGA data, BRD4 expression was significantly higher in tumor group than normal group (Figures 11C,D). The results suggested that high expression of BRD4 predicted poor prognosis in GBM.

## Discussion

BET proteins, and in particular BRD4, have been implicated in human disease especially cancer. The BET family directly regulates the MYC gene expression, potentially reducing cell proliferation (Beroukhim et al., 2010). Neurogenic cancers may be associated with differential expression of BET proteins including medulloblastoma and neuroblastoma (Puissant et al., 2013). BET protein is also necessary for the proliferation of glioblastoma cells, BRD2 and BRD4 mRNA are significantly overexpressed in glioblastoma cells (Pastori et al., 2014). BRD2 and BRD4 are overexpressed in human primary and metastatic melanomas, and their inhibition results in downregulation of IL-6 and IL-8 (Klein et al., 2016). Many proteins that exploit BET proteins to recruit to specific regulatory complexes have been implicated in cancer development (Barbieri et al., 2013; Filippakopoulos and Knapp, 2014). In recent years, the role of BRD4 in brain glioma has been gradually concerned, but the research and application of BRD4 inhibitors in brain glioma are still limited. The effectiveness of GBM treatment is limited by the blood brain barrier and resistance to single drug (Yang et al., 2021). At present, OTX015 (MK-8628), a novel BRD2/3/4 inhibitor, used to treat glioma in preclinical trials, but the therapeutic effect of BET inhibitor on glioma needs further study. Glioma has a strong heterogeneity, and the effect of a single small molecule drug on glioma is limited. Therefore, the combination of BRD4 inhibitors and other drugs, optimization of dosage form are expected to be effective strategy to improve the therapeutic effect of BRD4 inhibition on glioma.

Recently, BRD4 has been reported to promote cell stemness (Wang et al., 2020) and progression in glioma (Kfoury et al., 2021). BRD4 was found to activate CLCF1 in U87 and U251 cells. Downregulation of CLCF1 significantly reduced cell proliferation, induced cell apoptosis and cell cycle G2 phase arrest, and weakened the migration and invasion in U87 and U251cells (Shen et al., 2022). In addition, GNE987 (BRD4 inhibitor) can damage the viability and inhibit cell proliferation in U87 cells, LN229 cells, U251 cells and A172 cells. GNE987 also can induce cell apoptosis, arrest cell cycle and promote apoptosis by down-regulating transcription of C-Myc and S100A16 (Ma et al., 2022). BRD4 inhibitor can inhibit the proliferation, migration and invasion of GBM cells and induce apoptosis. However, the exact mechanism of BRD4 in the progression of GBM remains unclear.

Immune components in the tumor microenvironment play an important role in clinical outcomes, and measurement of these immune components predicts long-term prognosis (Cooper et al., 2012; Yoshihara et al., 2013; Senbabaoglu et al., 2016). The differential expression of immune-related genes (IRGs) has been considered as an effective biomarker in many types of cancer (Schneider et al., 2019). Tumor microenvironment contains many subsets of immune cells with anti-tumor or tumor-promoting activity. (Hanahan and Weinberg, 2011). In addition, different tumor infiltrating immune cells will affect the prognosis of patients (Fridman et al., 2017). Therefore, IRGs can be used to identify potentially high-risk patients, thereby providing strategies for individualized treatment.

This study used bioinformatics analysis tools to screen tumor data from public databases for target gene analysis, and provided multiple layers of evidence for the potential of BRD4 as a molecular marker in GBM. The results also showed that the expression of BET genes were closely related to that of immune checkpoint gene in GBM. The level of BRD4 expression was significantly correlated with the tumor immune microenvironment in GBM patients. Overall, the expression of BRD4, one of BET genes, may play an important role in tumor development by participating in immune response. The over-expression of BRD4 may be used as molecular marker to identify high-risk subgroups of GBM patients. This study also analyzed the mechanism of BRD4 in GBM. On the one hand, it might be that p53 regulated downstream transcription factors through mTOR and MAPK signaling pathways, thereby affected cell cycle progression and inhibited tumor growth and metastasis. On the other hand, it might play a dual role in immune response or immune activation by regulating the regulatory T-cell in the tumor immune microenvironment to affect tumor progression.

## Conclusion

The upregulated BRD4 expression was illustrated in this study, BRD4 could be significant for immune infiltration and be valuable in guidelines for evaluation of prognosis in GBM patients. Therefore, this study suggested that BRD4 may be a valuable prognostic biomarker, and a potential target of precision therapy against GBM. However, There are still limitations in our study, and relevant *in vivo* experiments should be carried out to verify our results. It is hoped that our results will provide researchers with new insights and this potential target may have clinical applications.

## Data Availability

The datasets presented in this study can be found in online repositories. The names of the repository/repositories and accession number(s) can be found in the article/Supplementary Material.

## Abbreviations

BET, Bromodomain and extra terminal domain; CNV, Copy number variation; DFS, Disease-free survival; GBM, Glioblastoma multiforme; GEPIA, Gene expression profiling interaction analysis; GO, Gene ontology; KEGG, Kyoto encyclopedia of genes and genomes; LGG, Lower grade glioma; MF, Molecular function; OS, Overall survival; PPI, Protein-protein interaction; SCNA, Somatic copy number alterations; TCGA, The cancer genome atlas; TIMER, Tumor immune estimation resource TILs: Tumor-infiltrating lymphocytes.

## References

[B1] Ballas Z. K. (2018). The 2018 nobel prize in physiology or medicine: An exemplar of bench to bedside in immunology. J. Allergy Clin. Immunol. 142 (6), 1752–1753. 10.1016/j.jaci.2018.10.021 30539724

[B2] Barbieri I. Cannizzaro E. Dawson M. A. (2013). Bromodomains as therapeutic targets in cancer. Brief. Funct. Genomics 12 (3), 219–230. 10.1093/bfgp/elt007 23543289

[B3] Barnholtz-Sloan J. S. Ostrom Q. T. Cote D. (2018). Epidemiology of brain tumors. Neurol. Clin. 36 (3), 395–419. 10.1016/j.ncl.2018.04.001 30072062

[B4] Bauer K. Berghoff A. S. Preusser M. Heller G. Zielinski C. C. Valent P. (2021). Degradation of BRD4-a promising treatment approach not only for hematologic but also for solid cancer. Am. J. Cancer Res. 11 (2), 530–545. 33575085 PMC7868748

[B5] Bechter O. Schöffski P. (2020). Make your best BET: The emerging role of BET inhibitor treatment in malignant tumors. Pharmacol. Ther. 208, 107479. 10.1016/j.pharmthera.2020.107479 31931101

[B6] Beroukhim R. Mermel C. H. Porter D. Wei G. Raychaudhuri S. Donovan J. (2010). The landscape of somatic copy-number alteration across human cancers. Nature 463, 899–905. 10.1038/nature08822 20164920 PMC2826709

[B7] Cancer Genome Atlas Network (2012a). Comprehensive molecular characterization of human colon and rectal cancer. Nature 487 (7407), 330–337. 10.1038/nature11252 22810696 PMC3401966

[B8] Cancer Genome Atlas Network (2012b). Comprehensive molecular portraits of human breast tumours. Nature 490 (7418), 61–70. 10.1038/nature11412 23000897 PMC3465532

[B9] Cancer Genome Atlas Research Network (2011). Integrated genomic analyses of ovarian carcinoma. Nature 474 (7353), 609–615. 10.1038/nature10166 21720365 PMC3163504

[B10] Cancer Genome Atlas Research Network (2015). The molecular taxonomy of primary prostate cancer. Cell 163 (4), 1011–1025. 10.1016/j.cell.2015.10.025 26544944 PMC4695400

[B11] Ceribelli M. Kelly P. N. Shaffer A. L. Wright G. W. Xiao W. Yang Y. (2014). Blockade of oncogenic IκB kinase activity in diffuse large B-cell lymphoma by bromodomain and extraterminal domain protein inhibitors. Proc. .Natl .Acad .Sci .USA. 31, 11365–11370. 10.1073/pnas.1411701111 PMC4128109 25049379

[B12] Charles N. A. Holland E. C. Gilbertson R. Glass R. Kettenmann H. (2012). The brain tumor micro-environment. Glia 60 (3), 502–514. 10.1002/glia.21264 22379614

[B13] Cooper L. A. Gutman D. A. Chisolm C. Appin C. Kong J. Rong Y. (2012). The tumor microenvironment strongly impacts master transcriptional regulators and gene expression class of glioblastoma. Am. J. Pathol. 180, 2108–2119. 10.1016/j.ajpath.2012.01.040 22440258 PMC3354586

[B14] Donati B. Lorenzini E. Ciarrocchi A. (2018). BRD4 and cancer: Going beyond transcriptional regulation. Mol. Cancer 17 (1), 164. 10.1186/s12943-018-0915-9 30466442 PMC6251205

[B15] Filippakopoulos P. Knapp S. (2014). Targeting bromodomains: Epigenetic readers of lysine acetylation. Nat. . Rev. Drug Disc. 13 (5), 337–356. 10.1038/nrd4286 24751816

[B16] Fridman W. H. Zitvogel L. Sautès-Fridman C. Kroemer G. (2017). The immune contexture in cancer prognosis and treatment. Nat. Rev. Clin. Oncol. 14, 717–734. 10.1038/nrclinonc.2017.101 28741618

[B17] Gao W. Li Y. Zhang T. Lu J. Pan J. Qi Q. (2022). Systematic analysis of chemokines reveals CCL18 is a prognostic biomarker in glioblastoma. J. Inflamm. Res. 15, 2731–2743. 10.2147/JIR.S357787 35509325 PMC9059990

[B18] Gao Z. Yuan T. Zhou X. Ni P. Sun G. Li P. (2018). Targeting BRD4 proteins suppresses the growth of NSCLC through downregulation of eIF4E expression. Cancer Biol. Ther. 19 (5), 407–415. 10.1080/15384047.2018.1423923 29333921 PMC5915014

[B19] Hanahan D. Weinberg R. A. (2011). Hallmarks of cancer: The next generation. Cell 144, 646–674. 10.1016/j.cell.2011.02.013 21376230

[B20] Hoadley K. A. Yau C. Wolf D. M. Cherniack A. D. Tamborero D. Ng S. (2014). Multiplatform analysis of 12 cancer types reveals molecular classification within and across tissues of origin. Cell 158 (4), 929–944. 10.1016/j.cell.2014.06.049 25109877 PMC4152462

[B21] Huang D. W. Sherman B. T. Lempicki R. A. (2009). Systematic and integrative analysis of large gene lists using DAVID bioinformatics resources. Nat. Protoc. 4 (1), 44–57. 10.1038/nprot.2008.211 19131956

[B22] Jin K. Qiu S. Jin D. Zhou X. Zheng X. Li J. (2021). Development of prognostic signature based on immune-related genes in muscle-invasive bladder cancer: Bioinformatics analysis of TCGA database. Aging (Albany NY) 13 (2), 1859–1871. 10.18632/aging.103787 33465047 PMC7880322

[B23] Kfoury N. Qi Z. Prager B. C. Wilkinson M. N. Broestl L. Berrett K. C. (2021). Brd4-bound enhancers drive cell-intrinsic sex differences in glioblastoma. Proc. Natl. Acad. Sci. U. S. A. 118 (16), e2017148118. 10.1073/pnas.2017148118 33850013 PMC8072233

[B24] Klein K. Kabala P. A. Grabiec A. M. Gay R. E. Kolling C. Lin L. L. (2016). The bromodomain protein inhibitor I-BET151 suppresses expression of inflammatory genes and matrix degrading enzymes in rheumatoid arthritis synovial fibroblasts. Ann. Rheum. Dis. 75 (2), 422–429. 10.1136/annrheumdis-2014-205809 25467295

[B25] Lapointe S. Perry A. Butowski N. A. (2018). Primary brain tumours in adults. Lancet 392 (10145), 432–446. 10.1016/S0140-6736(18)30990-5 30060998

[B26] Li R. Wang H. Liang Q. Chen L. Ren J. (2022). Radiotherapy for glioblastoma: Clinical issues and nanotechnology strategies. Biomater. Sci. 10 (4), 892–908. 10.1039/d1bm01401c 34989724

[B27] Ma L. Li G. Yang T. Zhang L. Wang X. Xu X. (2022). An inhibitor of BRD4, GNE987, inhibits the growth of glioblastoma cells by targeting C-Myc and S100A16. Cancer Chemother. Pharmacol. 90 (6), 431–444. 10.1007/s00280-022-04483-7 36224471 PMC9637061

[B28] Niedbała M. Malarz K. Sharma G. Kramer-Marek G. Kaspera W. (2022). Glioblastoma: Pitfalls and opportunities of immunotherapeutic combinations. Onco Targets Ther. 15, 437–468. 10.2147/OTT.S215997 35509452 PMC9060812

[B29] Pastori C. Daniel M. Penas C. Volmar C. H. Johnstone A. L. Brothers S. P. (2014). BET bromodomain proteins are required for glioblastoma cell proliferation. Epigenetics 9, 611–620. 10.4161/epi.27906 24496381 PMC4121371

[B30] Pervaiz M. Mishra P. Gunther S. (2018). Bromodomain drug discovery the past, the present, and the future. Chem. Rec. 18 (12), 1808–1817. 10.1002/tcr.201800074 30289209

[B31] Puissant A. Frumm S. M. Alexe G. Bassil C. F. Qi J. Chanthery Y. H. (2013). Targeting MYCN in neuroblastoma by BET bromodomain inhibition. Cancer Discov. 3, 308–323. 10.1158/2159-8290.CD-12-0418 23430699 PMC3672953

[B32] Ravi R. Noonan K. A. Pham V. Bedi R. Zhavoronkov A. Ozerov I. V. (2018). Bifunctional immune checkpoint-targeted antibody-ligand traps that simultaneously disable TGFβ enhance the efficacy of cancer immunotherapy. Nat. Commun. 9 (1), 741. 10.1038/s41467-017-02696-6 29467463 PMC5821872

[B33] Rhodes D. R. Yu J. Shanker K. Deshpande N. Varambally R. Ghosh D. (2004). Oncomine: A cancer microarray database and integrated data-mining platform. Neoplasia 6 (1), 1–6. 10.1016/s1476-5586(04)80047-2 15068665 PMC1635162

[B34] Schneider A. K. Chevalier M. F. Derré L. (2019). The multifaceted immune regulation of bladder cancer. Nat. Rev. Urol. 16, 613–630. 10.1038/s41585-019-0226-y 31501534

[B35] Şenbabaoglu Y. Gejman R. S. Winer A. G. Liu M. Van Allen E. M. de Velasco G. (2016). Tumor immune microenvironment characterization in clear cell renal cell carcinoma identifies prognostic and immunotherapeutically relevant messenger RNA signatures. Genome Biol. 17, 231. 10.1186/s13059-016-1092-z 27855702 PMC5114739

[B36] Shen S. H. Guo J. F. Huang J. Zhang Q. Cui Y. (2022). Bromodomain-containing protein 4 activates cardiotrophin-like cytokine factor 1, an unfavorable prognostic biomarker, and promotes glioblastoma *in vitro* . Ann. Transl. Med. 10 (8), 475. 10.21037/atm-22-1164 35571403 PMC9096406

[B37] Shi J. Vakoc C. R. (2014). The mechanisms behind the therapeutic activity of BET bromodomain inhibition. Mol. Cell 54 (5), 728–736. 10.1016/j.molcel.2014.05.016 24905006 PMC4236231

[B38] Sjostedt E. Zhong W. Fagerberg L. Karlsson M. Mitsios N. Adori C. (2020). An atlas of the protein-coding genes in the human, pig, and mouse brain. Science 367 (6482), eaay5947. 10.1126/science.Aay5947 32139519

[B39] Tang Z. F. Li C. W. Kang B. Gao G. Li C. Zhang Z. (2017). Gepia: A web server for cancer and normal gene expression profiling and interactive analyses. Nucleic Acids Res. 45 (1), W98–W102. 10.1093/nar/gkx247 28407145 PMC5570223

[B40] Vasaikar S. V. Straub P. Wang J. Zhang B. (2018). LinkedOmics: Analyzing multi-omics data within and across 32 cancer types. Nucleic Acids Res. 46 (1), D956–D963. 10.1093/nar/gkx1090 29136207 PMC5753188

[B41] Wang J. Quan Y. Lv J. Gong S. Dong D. (2020). BRD4 promotes glioma cell stemness via enhancing miR-142-5p-mediated activation of Wnt/β-catenin signaling. Environ. Toxicol. 35, 368–376. 10.1002/tox.22873 31724259

[B42] Wang J. Sun J. Liu L. N. Flies D. B. Nie X. Toki M. (2019). Siglec-15 as an immune suppressor and potential target for normalization cancer immunotherapy. Nat. Med. 25 (4), 656–666. 10.1038/s41591-019-0374-x 30833750 PMC7175920

[B43] Wesseling P. Capper D. (2018). WHO 2016 classification of gliomas. Neuropathol. Appl. Neurobiol. 44 (2), 139–150. 10.1111/nan.12432 28815663

[B44] Wu W. Klockow J. L. Zhang M. Lafortune F. Chang E. Jin L. (2021). Glioblastoma multiforme (GBM): An overview of current therapies and mechanisms of resistance. Pharmacol. Res. 171, 105780. 10.1016/j.phrs.2021.105780 34302977 PMC8384724

[B45] Yang H. Wei L. Xun Y. Yang A. You H. (2021). BRD4: An emerging prospective therapeutic target in glioma. Mol. Ther. Oncolytics 21, 1–14. 10.1016/j.omto.2021.03.005 33851008 PMC8010576

[B46] Yoshihara K. Shahmoradgoli M. Martínez E. Vegesna R. Kim H. Torres-Garcia W. (2013). Inferring tumour purity and stromal and immune cell admixture from expression data. Nat. Commun. 4, 2612. 10.1038/ncomms3612 24113773 PMC3826632

[B47] Zhang Z. Lin E. Zhuang H. Xie L. Feng X. Liu J. (2020). Construction of a novel gene-based model for prognosis prediction of clear cell renal cell carcinoma. Cancer Cell Int. 20, 27. 10.1186/s12935-020-1113-6 32002016 PMC6986036

[B48] Zhou Y. Zhou B. Pache L. Chang M. Khodabakhshi A. H. Tanaseichuk O. (2019). Metascape provides a biologist-oriented resource for the analysis of systems-level datasets. Nat. Commun. 10 (1), 1523. 10.1038/s41467-019-09234-6 30944313 PMC6447622

