# Supplementary material for: Comprehensive analysis of the prognosis and immune infiltrates for the BET protein family reveals the significance of BRD4 in glioblastoma multiforme
Source: Front Cell Dev Biol. 2023 Jan 12;11:1042490. doi: 10.3389/fcell.2023.1042490 (PMC9878708; doi:10.3389/fcell.2023.1042490)
Supplement: Supplementary file 1 [file Table1.PDF]

TABLES

TABLE 1. Disease-gene association enrichment analysis of BET genes in DisGeNET (Metascape)

GO	Description	Count	(%)	Log10 (P)	Log10 (q)
C0205833	Medullomyoblastoma	3	60	-7.60	-3.40
C1275668	Melanotic medulloblastoma	3	60	-7.60	-3.40
C0751291	Desmoplastic Medulloblastoma	3	60	-7.30	-3.20
C0278701	Gastric Adenocarcinoma	3	60	-4.50	-0.57
C0278876	Adult Medulloblastoma	3	60	-3.80	-0.06
C0278510	Childhood Medulloblastoma	3	60	-3.80	-0.06

Note: "Log10(P)" is the p-value in log base 10. "Log10(q)" is the multi-test adjusted p-value in log base10.

TABLE 2. GO enrichment items of the differentiated BET genes (Metascape)

GO	Category	Description	Count	(%)	Log10 (P)	Log10 (q)
GO:0006325	GO Biological Processes	chromatin organization	5	100.00	-8.47	-4.13
GO:0006338	GO Biological Processes	chromatin remodeling	4	80.00	-7.34	-3.30

Note: "Log10(P)" is the p-value in log base 10. "Log10(q)" is the multi-test adjusted p-value in log base10.

TABLE 3. The top 10 gene expression correlation analysis for BRD4 (LinkedOmics)

Target Gene/Attribute	Spearman Correlation	P-value	FDR	Event_SD

Target Gene/Attribute	Spearman Correlation	P-value	FDR	Event_SD
BRD4	1.000	1.000e-48	1.000e-47	528
WIZ	0.7719	1.000e-48	1.000e-47	528
KHSRP	0.7555	1.000e-48	1.000e-47	528
CABIN1	0.7244	1.000e-48	1.000e-47	528
GNA11	0.7237	1.000e-48	1.000e-47	528
SMARCA4	0.7235	1.000e-48	1.000e-47	528
KIAA0515	0.7208	1.000e-48	1.000e-47	528
C19orf29	0.7118	1.000e-48	1.000e-47	528
GATAD2A	0.7087	1.000e-48	1.000e-47	528
PIP5K1C	0.7044	1.000e-48	1.000e-47	528

TABLE 4. The top 10 functional roles of biological processes for BRD4

Gene Set	Description	Leading Edge Number	Normalized Enrichment Score(NES)	P Value	FDR
GO:0030705	cytoskeleton-dependent intracellular transport	49	1.5000	0	0.056516
GO:0006397	mRNA processing	132	1.4911	0	0.040821
GO:0016197	endosomal transport	59	1.4813	0	0.039064
GO:1902115	regulation of organelle assembly	58	1.4792	0	0.038966

GO:0016072	rRNA metabolic process	69	1.4379	0	0.065012
GO:0016569	covalent chromatin modification	124	1.4365	0	0.063479
GO:0051169	nuclear transport	108	1.4298	0	0.064635
GO:0010608	posttranscriptional regulation of gene expression	123	1.4030	0	0.064074
GO:0002526	acute inflammatory response	48	-1.6802	0	0.063072
GO:0009593	detection of chemical stimulus	56	-2.3212	0	0

TABLE 5. The top 10 functional roles of cellular components(CC) for BRD4

**Gene Set**	**Description**	**Leading Edge Number**	**Normalized Enrichment Score(NES)**	**P Value**	**FDR**
GO:0005681	spliceosomal complex	53	1.5144	0	0.011015
GO:0034399	nuclear periphery	54	1.4904	0	0.016890
GO:0016607	nuclear speck	118	1.4700	0	0.017951
GO:0000151	ubiquitin ligase complex	65	1.4575	0	0.019736
GO:0005802	trans-Golgi network	51	1.3911	0	0.033182
GO:0044452	nucleolar part	38	1.3902	0	0.031273
GO:0048475	coated membrane	28	1.3838	0	0.032868
GO:0000790	nuclear chromatin	94	1.3810	0	0.033161

GO:0072562	blood microparticle	28	-1.3956	0	0.14986
GO:0031225	anchored component of membrane	48	-1.7850	0	0.028342

TABLE 6.The top 10 functional roles of molecular functions(MF) for BRD4

**Gene Set**	**Description**	**Leading Edge Number**	**Normalized Enrichment Score(NES)**	**P Value**	**FDR**
GO:0042393	histone binding	64	1.5023	0	0.019699
GO:0017048	Rho GTPase binding	46	1.4887	0	0.018005
GO:0032182	ubiquitin-like protein binding	38	1.4721	0	0.022238
GO:0004386	helicase activity	59	1.4646	0	0.022860
GO:0017137	Rab GTPase binding	42	1.4485	0	0.026992
GO:0003729	mRNA binding	67	1.4485	0	0.028413
GO:0019787	ubiquitin-like protein transferase activity	94	1.4405	0	0.028958
GO:0004896	cytokine receptor activity	28	-1.9179	0	0.021093
GO:0004497	monooxygenase activity	39	-1.9626	0	0.017718
GO:0004984	olfactory receptor activity	32	-2.4443	0	0

TABLE 7. The top 19 KEGG pathway analysis for BRD4

Gene Set	Description	Leading Edge Number	Normalized Enrichment Score(NES)	P Value	FDR
hsa04520	Adherens junction	32	1.4821	0	0.085886
hsa03015	mRNA surveillance pathway	35	1.461	0	0.08834
hsa03040	Spliceosome	41	1.4313	0	0.10049
hsa03008	Ribosome biogenesis in eukaryotes	20	1.4071	0	0.10217
hsa04120	Ubiquitin mediated proteolysis	51	1.4064	0	0.094338
hsa04330	Notch signaling pathway	13	1.4031	0.010121	0.081687
hsa05210	Colorectal cancer	24	1.4025	0.0020121	0.077502
hsa04152	AMPK signaling pathway	36	1.3946	0	0.081523
hsa04137	Mitophagy	23	1.3797	0.010142	0.098973
hsa05214	Glioma	18	1.371	0.0040241	0.097678
hsa00190	Oxidative phosphorylation	29	-1.5586	0	0.089424
hsa04610	Complement and coagulation cascades	24	-1.6045	0	0.068344
hsa00053	Ascorbate and aldarate metabolism	3	-1.6884	0	0.040316
hsa04742	Taste transduction	27	-1.7642	0	0.030055

hsa00601	Glycosphingolipid biosynthesis	11	-1.8008	0	0.0243
hsa00591	Linoleic acid metabolism	8	-1.8143	0.022222	0.021441
hsa04950	Maturity onset diabetes of the young	9	-2.1016	0	0
hsa05330	Allograft rejection	14	-2.2568	0	0
hsa05204	Chemical carcinogenesis	22	-2.5317	0	0
