# Supplementary figures and images for: Comprehensive analysis of the prognosis and immune infiltrates for the BET protein family reveals the significance of BRD4 in glioblastoma multiforme
Source: Front Cell Dev Biol. 2023 Jan 12;11:1042490. doi: 10.3389/fcell.2023.1042490 (PMC9878708; doi:10.3389/fcell.2023.1042490)

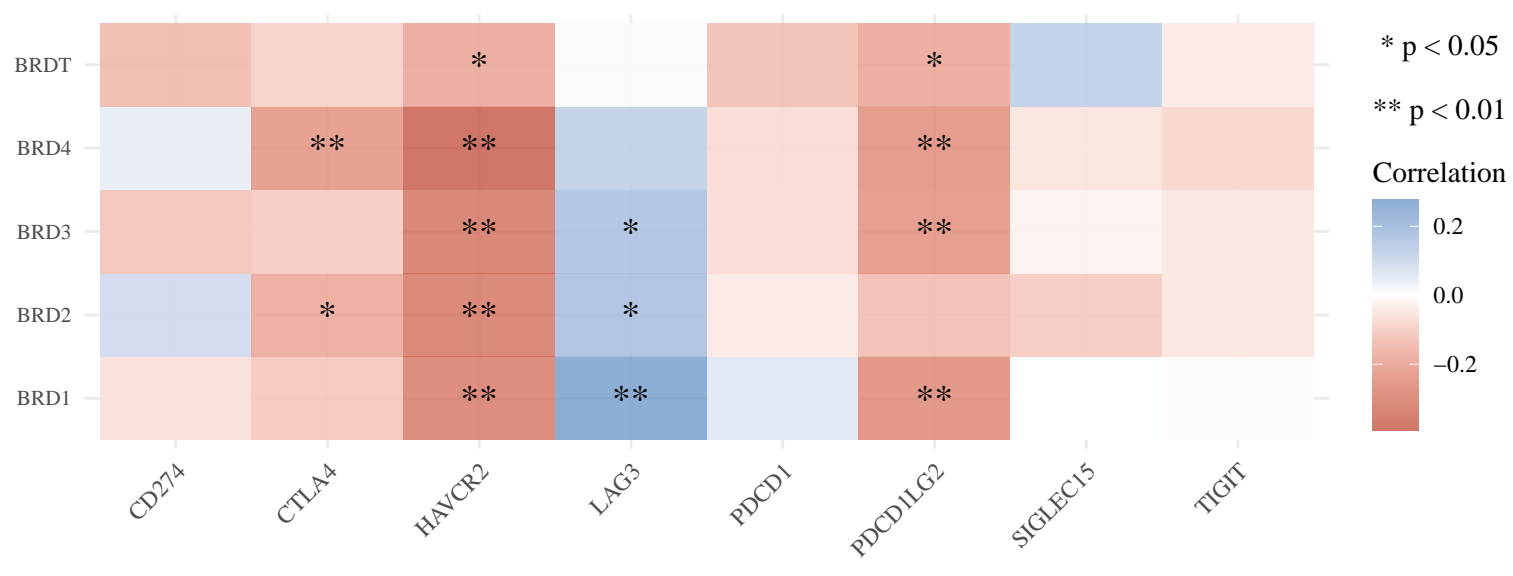

Supplement: Supplementary file 2 [file DataSheet1.ZIP › Data Sheet/Heatmap of the correlation between BET and immune checkpoint/Heatmap of the correlation between BET and immune checkpoint .pdf]

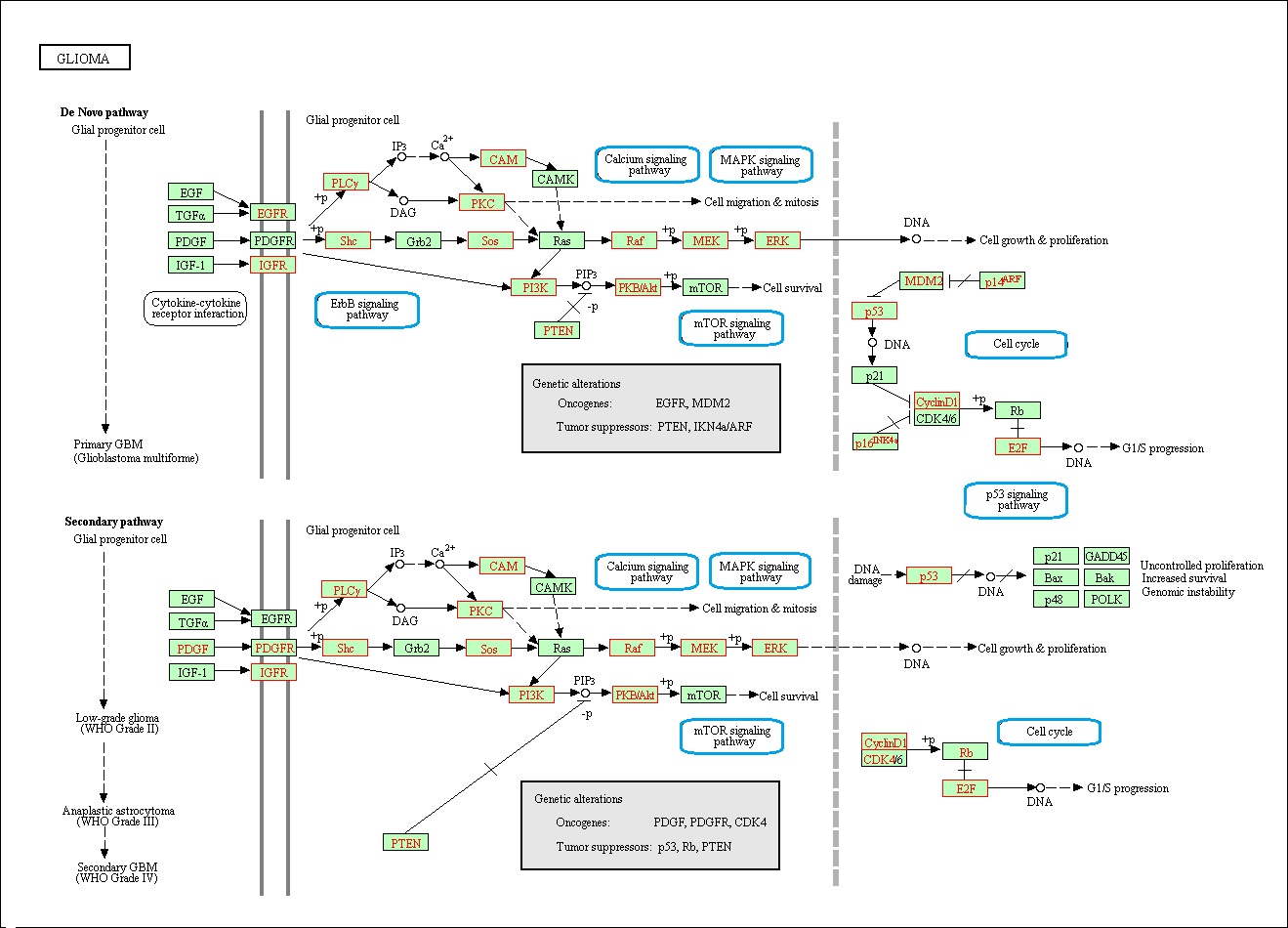

Supplement: Supplementary file 2 [file DataSheet1.ZIP › Data Sheet/LinkedOmics data/Glioma pathway.jpg]

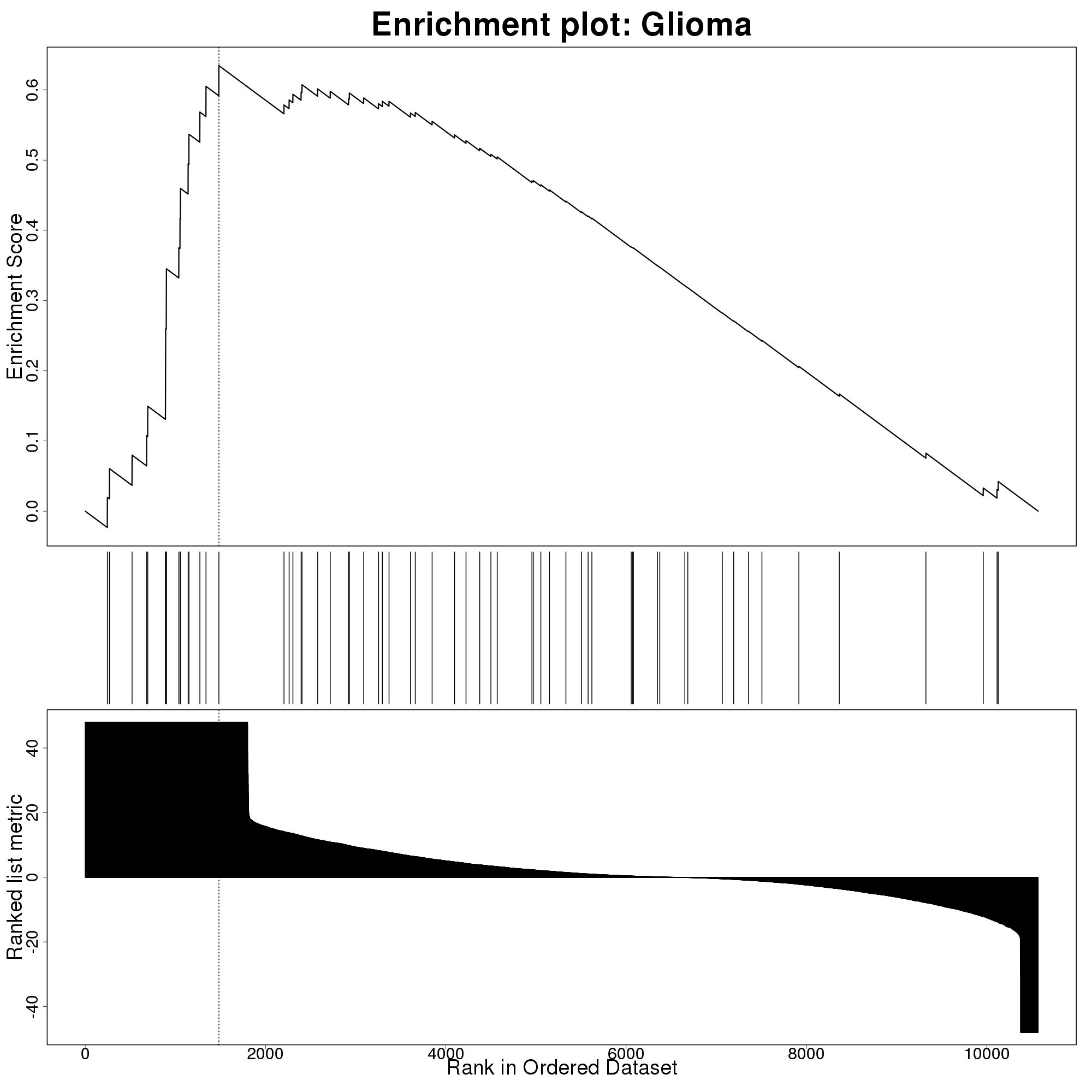

Supplement: Supplementary file 2 [file DataSheet1.ZIP › Data Sheet/LinkedOmics data/hsa05214.png]

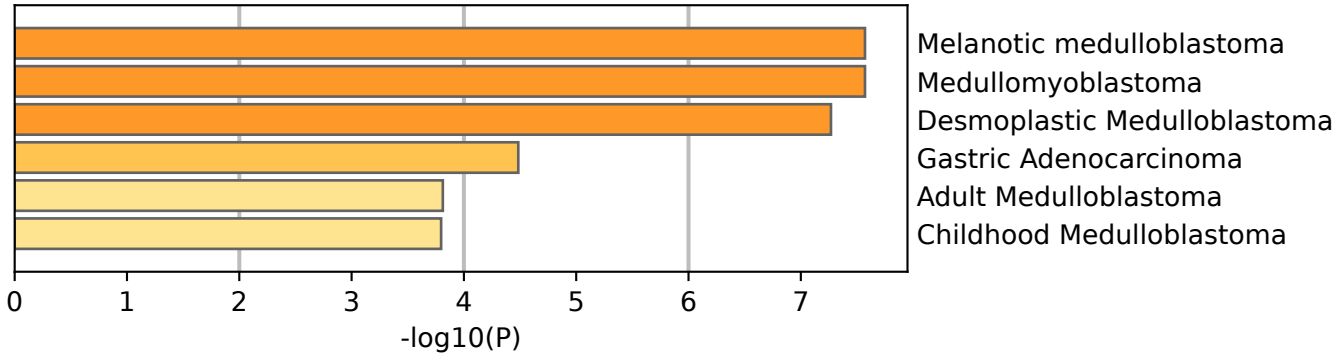

Supplement: Supplementary file 2 [file DataSheet1.ZIP › Data Sheet/Metascape data/Enrichment_QC/HeatmapSelectedGO_DisGeNET.pdf]

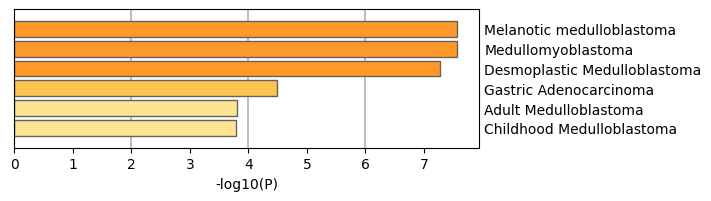

Supplement: Supplementary file 2 [file DataSheet1.ZIP › Data Sheet/Metascape data/Enrichment_QC/HeatmapSelectedGO_DisGeNET.png]

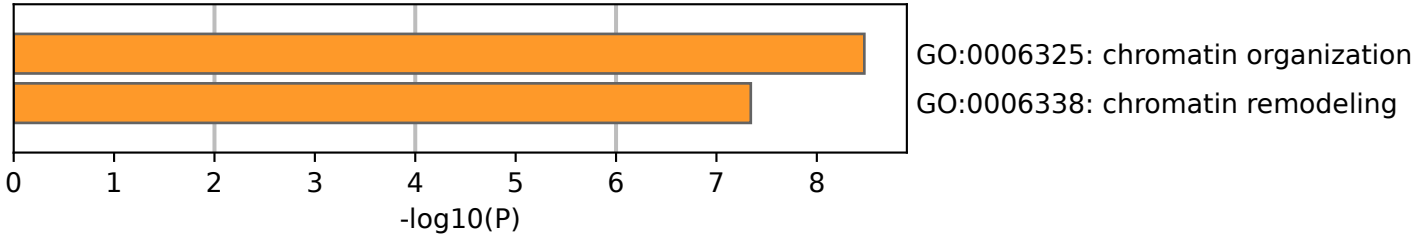

Supplement: Supplementary file 2 [file DataSheet1.ZIP › Data Sheet/Metascape data/Enrichment_heatmap/HeatmapSelectedGO.pdf]

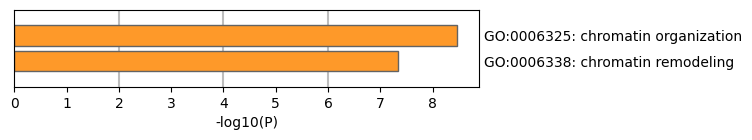

Supplement: Supplementary file 2 [file DataSheet1.ZIP › Data Sheet/Metascape data/Enrichment_heatmap/HeatmapSelectedGO.png]

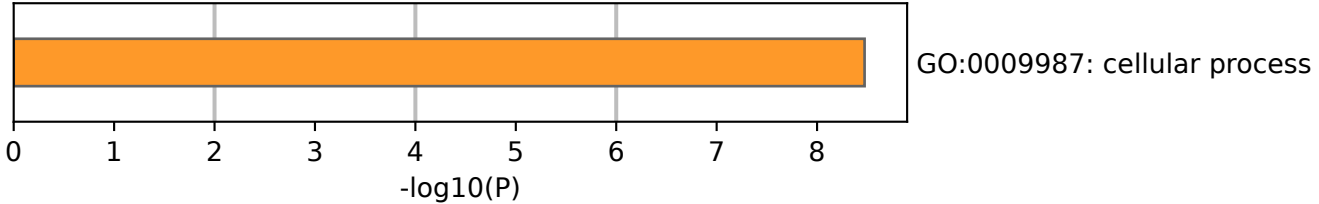

Supplement: Supplementary file 2 [file DataSheet1.ZIP › Data Sheet/Metascape data/Enrichment_heatmap/HeatmapSelectedGOParent.pdf]

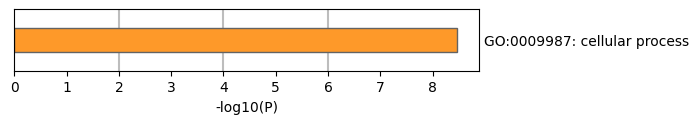

Supplement: Supplementary file 2 [file DataSheet1.ZIP › Data Sheet/Metascape data/Enrichment_heatmap/HeatmapSelectedGOParent.png]

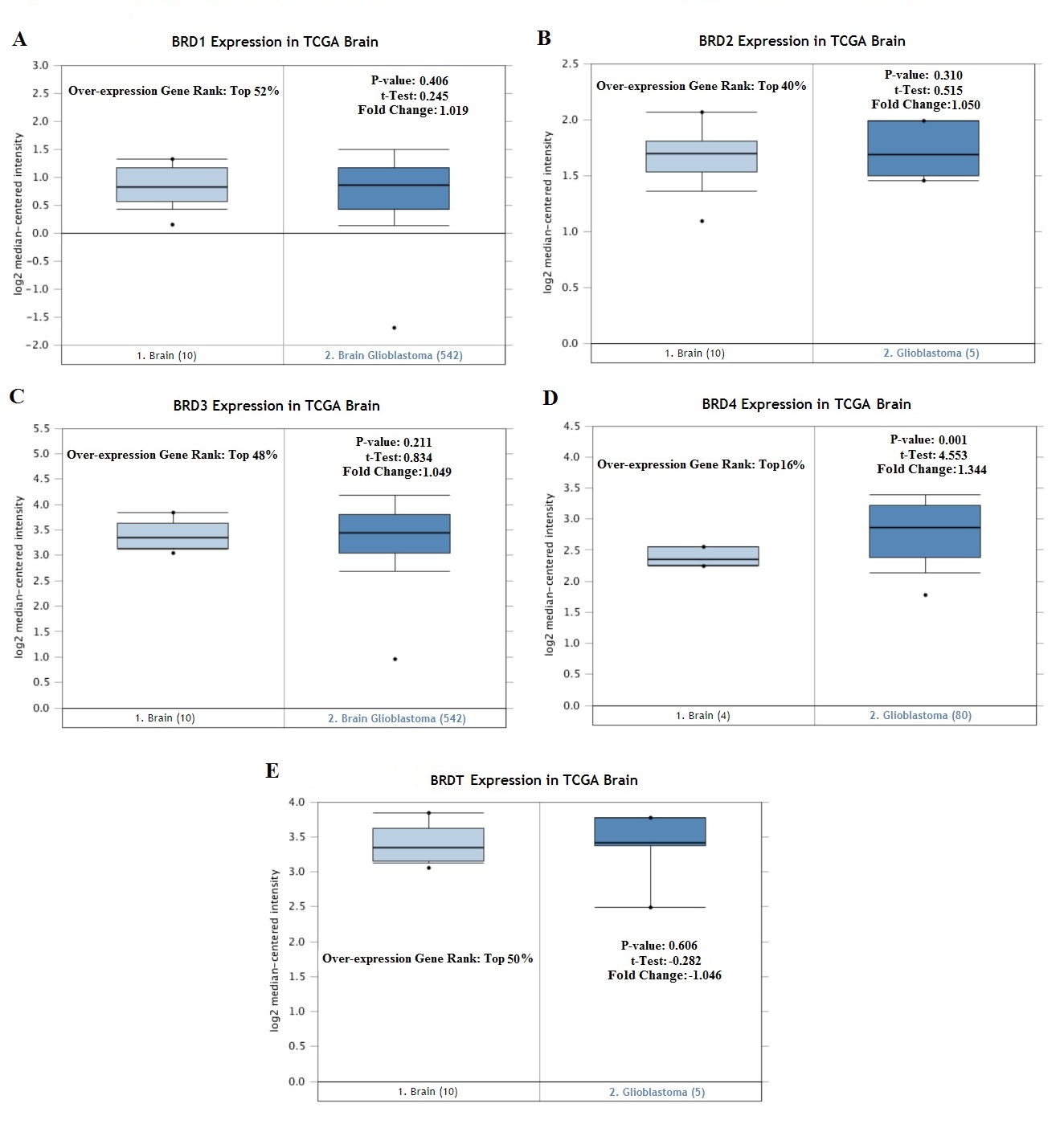

Supplement: Supplementary file 4 [file DataSheet2.ZIP › Supplementary Material/Supplementary Image/FIGURE S1 .jpg]

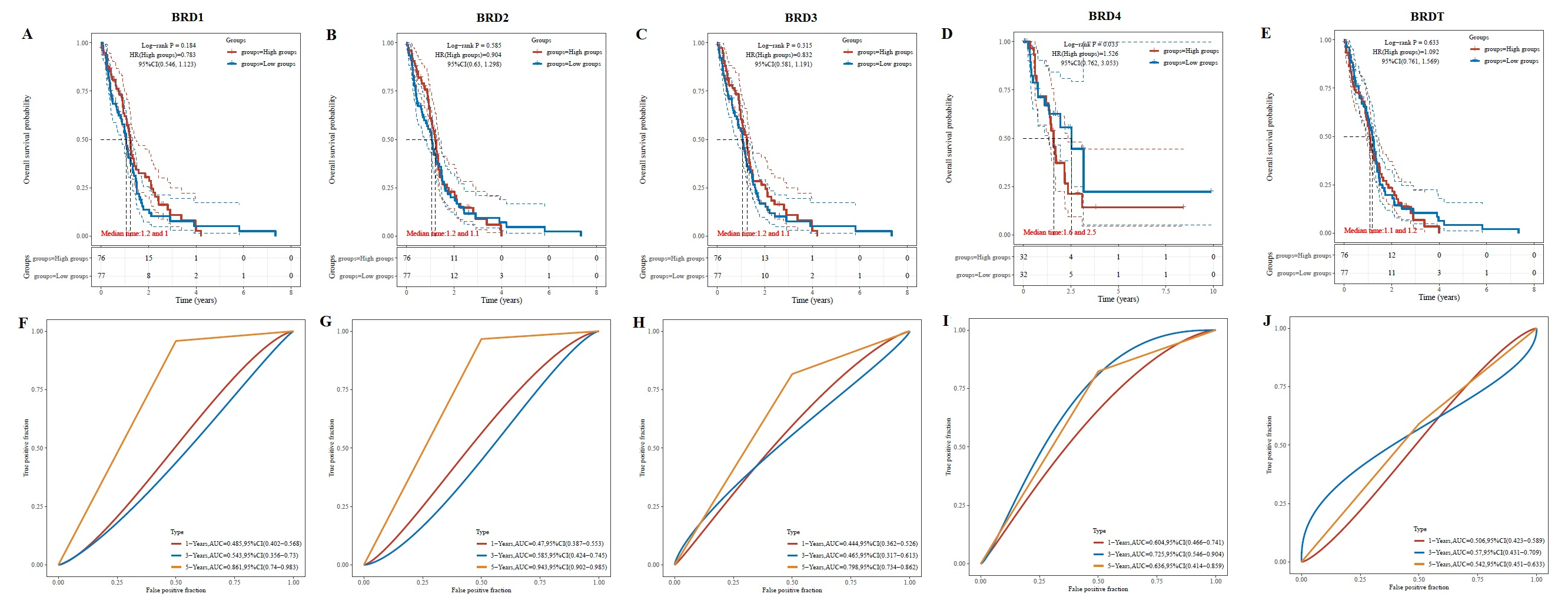

Supplement: Supplementary file 4 [file DataSheet2.ZIP › Supplementary Material/Supplementary Image/FIGURE S2.jpg]

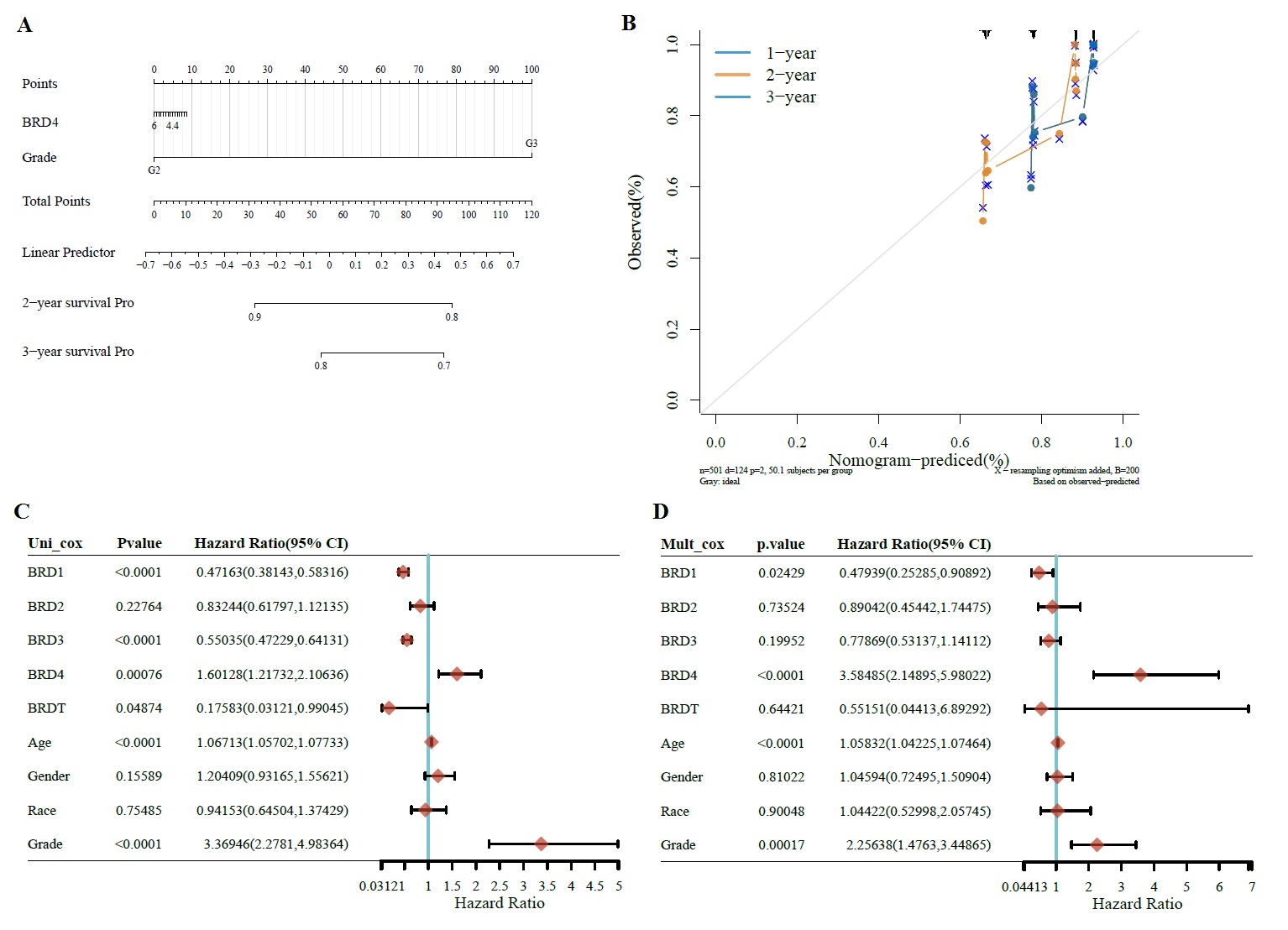

Supplement: Supplementary file 4 [file DataSheet2.ZIP › Supplementary Material/Supplementary Image/FIGURE S3.jpg]
