# Supplementary material for: Comprehensive analysis of the prognosis and immune infiltrates for the BET protein family reveals the significance of BRD4 in glioblastoma multiforme
Source: Front Cell Dev Biol. 2023 Jan 12;11:1042490. doi: 10.3389/fcell.2023.1042490 (PMC9878708; doi:10.3389/fcell.2023.1042490)
Supplement: Supplementary file 3 [file DataSheet3.PDF]

FIGURES

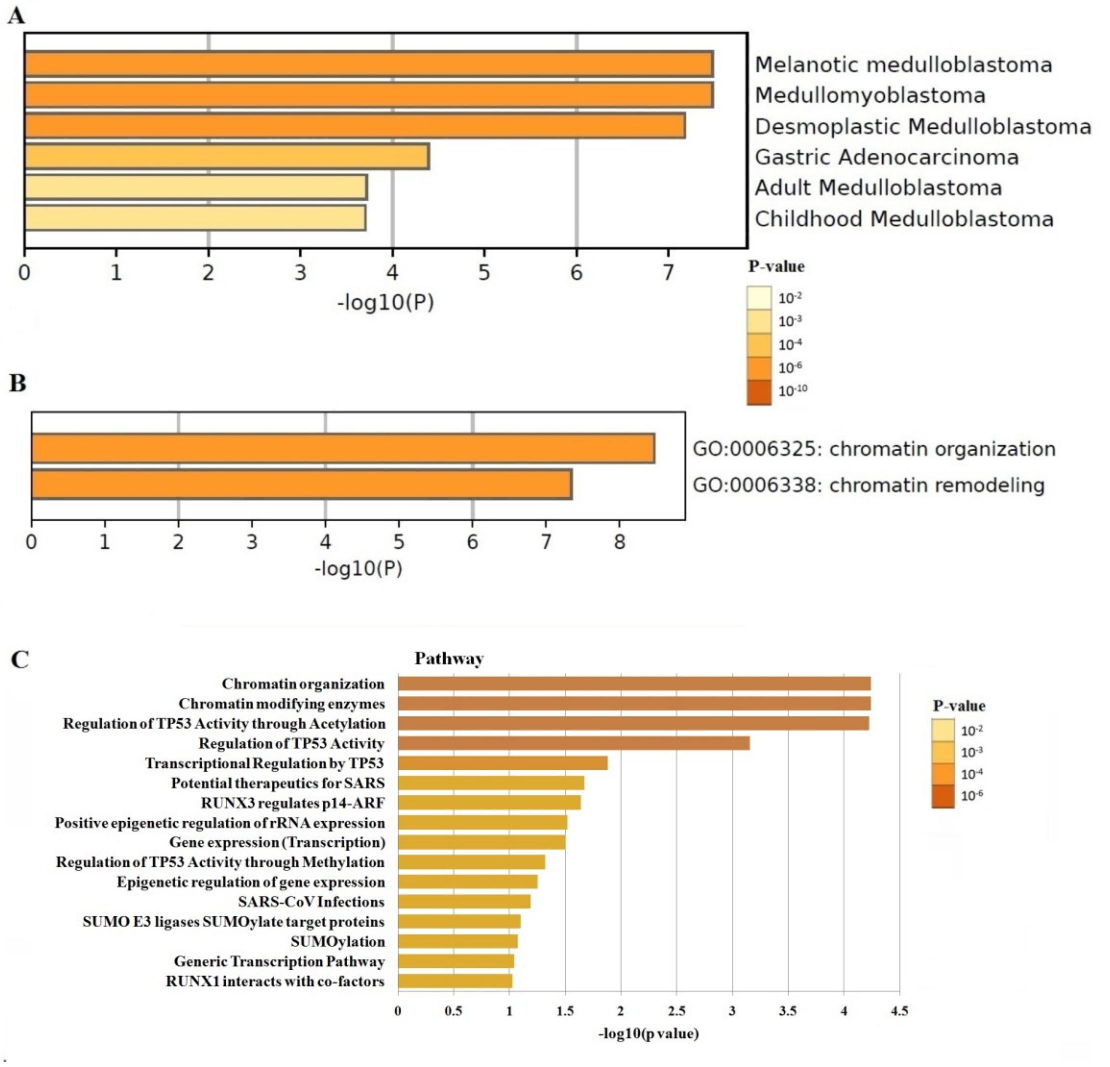

FIGURE 1. The enrichment analysis of BET gene was predicted by bioinformatics analysis. **(A)** The top few enriched clusters were identified in DisGeNET ontology categories in Metascape database. **(B)** The Go enrichment heatmap was plotted with p-value in Metascape database. **(C)** The Reactome pathway enrichment analysis of BET co-expression genes in David database. The more abundant the gene, the darker the color.

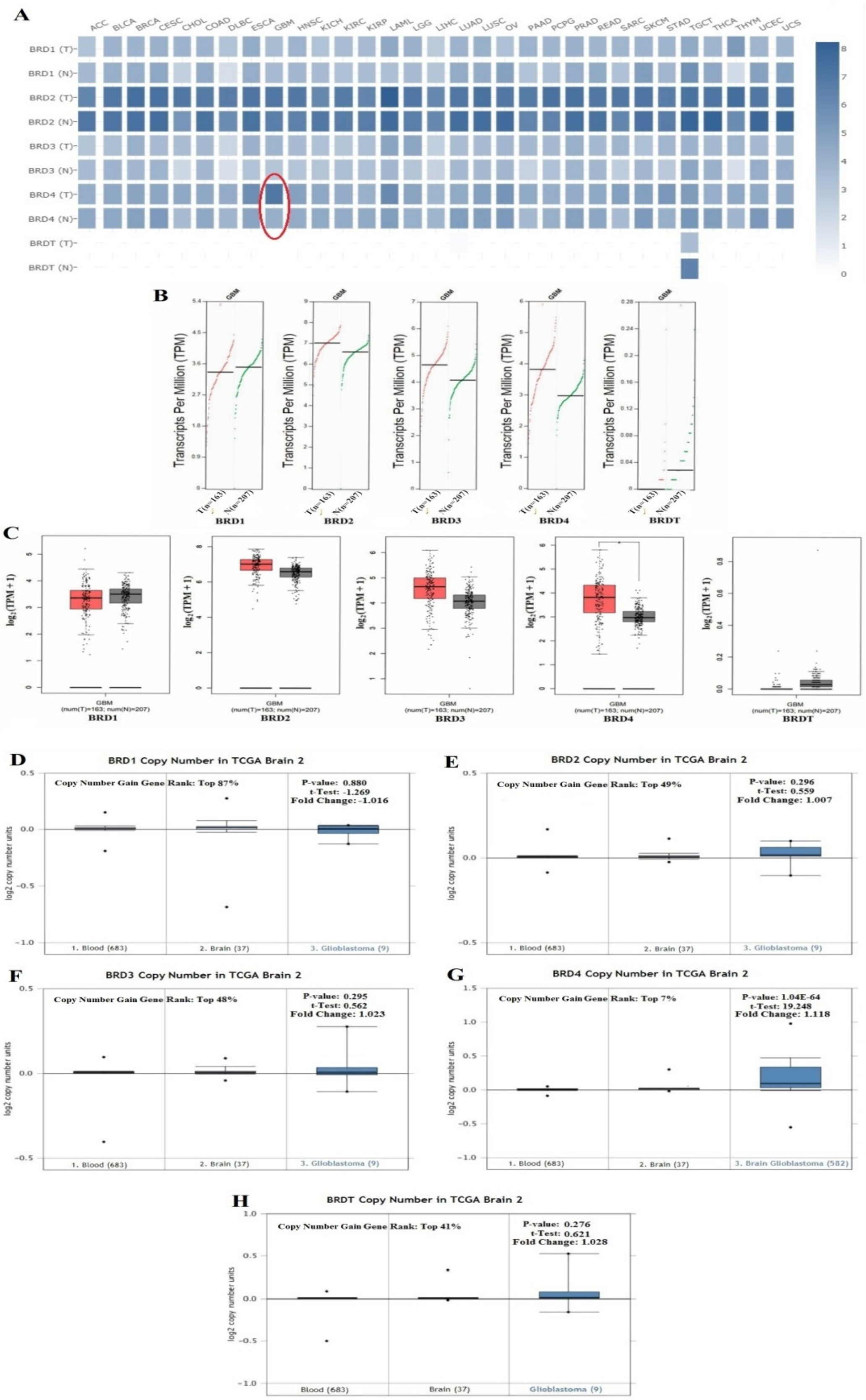

FIGURE 2. The transcription levels of BET genes in GBM samples. **(A)** Multiple gene comparison in different cancer types were generated in GEPIA database. T:Tumor tissues; N: Normal tissues. **(B)** The transcription levels of BET genes in

GBM patients. The scatter diagram of expression of BET genes in GBM. **(C)** The box plot of BET genes expression in GEPIA database（*P＜0.05）. **(D-H)** The copy number of BRD1, BRD2, BRD3, BRD4, BRDT were shown in TCGA Brain 2 cohort, respectively. The p-values, t-Test, fold change, and copy number gain gene rank were based on Oncomine database.

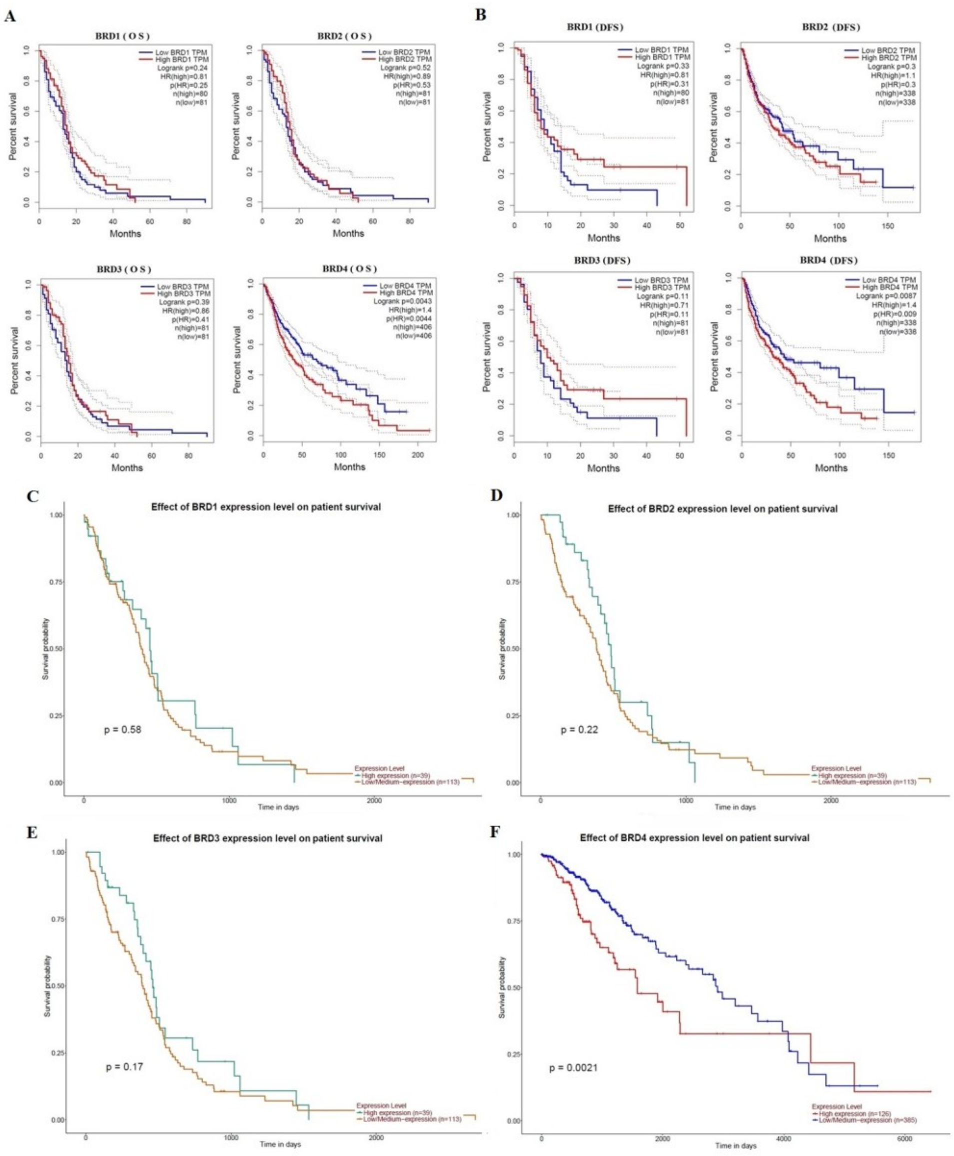

FIGURE 3. The prognostic value of BET genes in GBM patients from the GEPIA database. The survival curves were plotted using the Kaplan–Meier Plotter . **(A)** The overall survival rate(OS) of BRD1, BRD2, BRD3 and BRD4 mRNA, respectively. **(B)**The disease-free survival rate(DFS）of BRD1, BRD2, BRD3 and BRD4 mRNA, respectively. **(C-F)** The effect of BRD1, BRD2, BRD3 and BRD4 expression level on patient survival from UALCAN database, respectively.

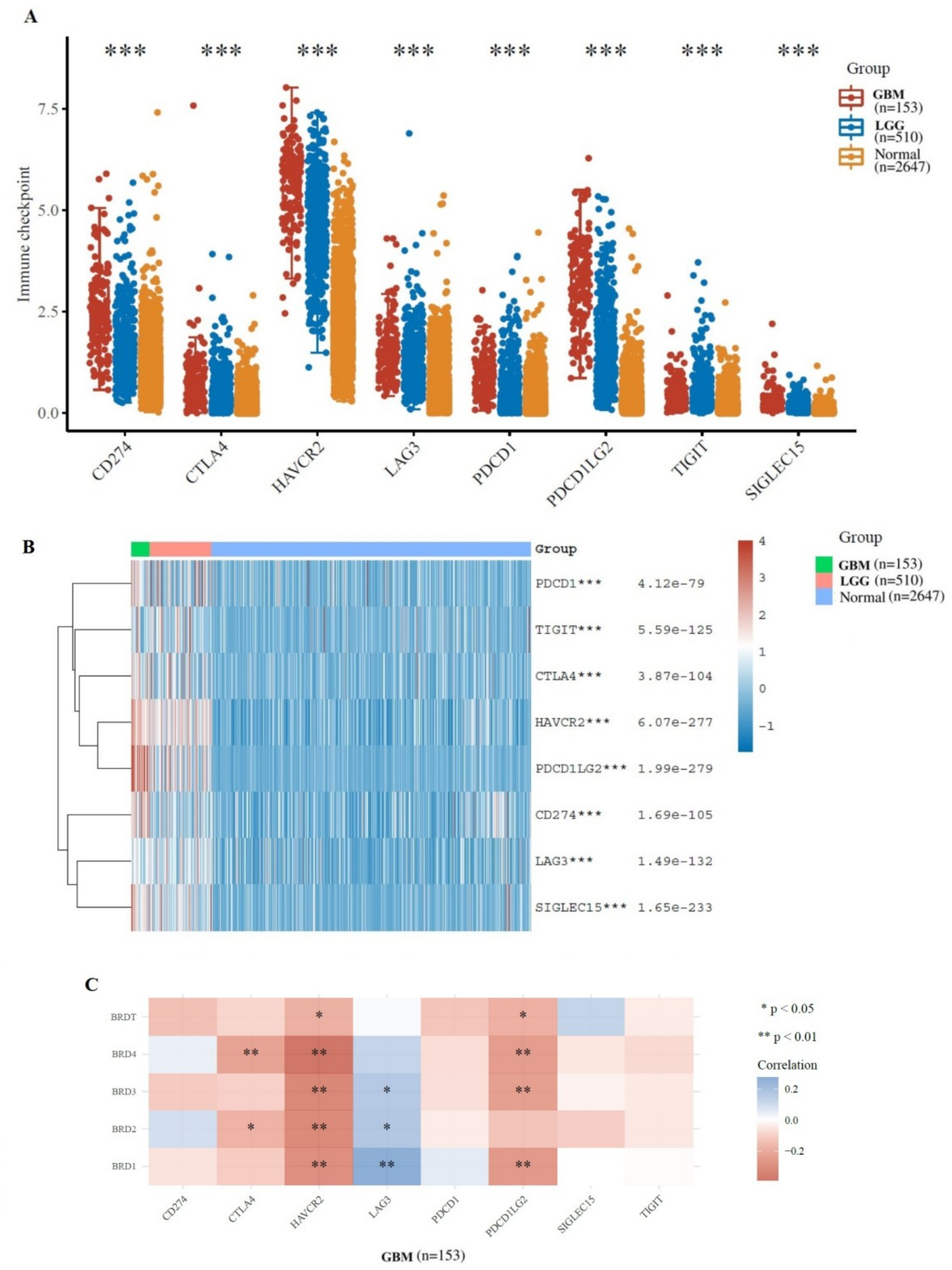

FIGURE 4. Immunological checkpoint related transcriptional level and expression in brain tumor samples in TCGA dataset. **(A)**The expression distribution of immune checkpoints gene (CD274, CTLA4, HAVCR2, LAG3, SIGLEC15, TIGIT, PDCD1 and PDCD1LG2) in tumor and normal tissues. **(B)**The heatmap of gene expression related to immune checkpoint(*p<0.05, **p<0.01, ***p<0.001). **(C)**The correlation between BET genes expression and immune checkpoint gene expression in GBM.

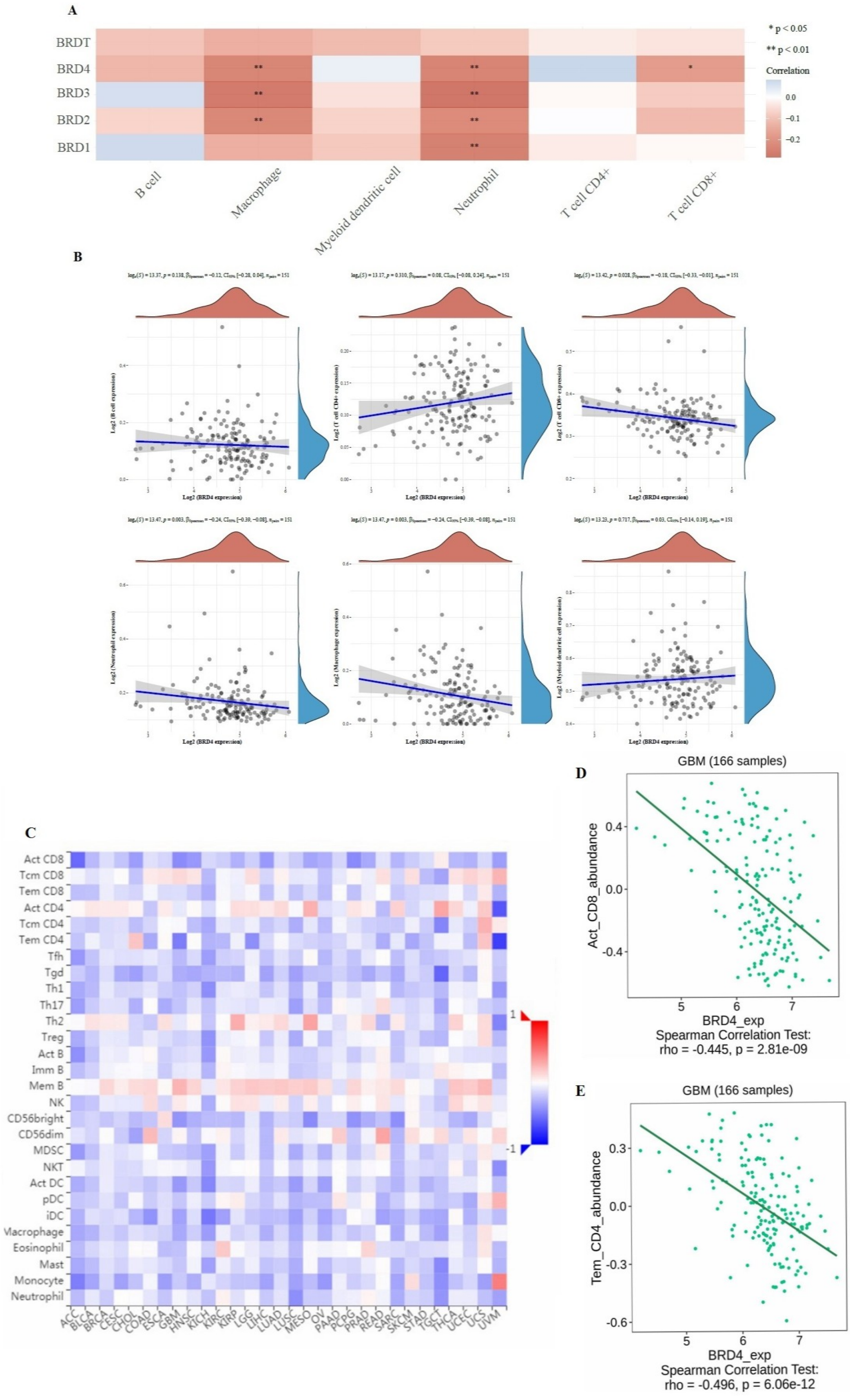

FIGURE 5. The correlation between BET genes expression and immune score was analyzed in TIMER and TISIDB database. **(A)** The heatmap of the correlation

between BET genes and immune score in six types of immune cells. The different colors indicated different correlation coefficients (blue for positive correlation and red for negative correlation). **(B)** Relationships between the immune infiltration abundances and BRD4 expression in different immune cells (B cells, CD4+T cells, CD8+T cells, neutrophils, macrophages and myeloid dendritic cells). **(C)** The correlations between expression of BRD4 and 28 types of TILs across human heterogeneous cancers. **(D)** BRD4 significantly negative correlated with abundance of activated CD8 T cell (Act_CD8; rho = -0.445, $p < 0.001$). **(E)** BRD4 significantly negative correlated with abundance of effector memeory CD4 T cell (Tem_CD4; rho = -0.496, $p < 0.001$).

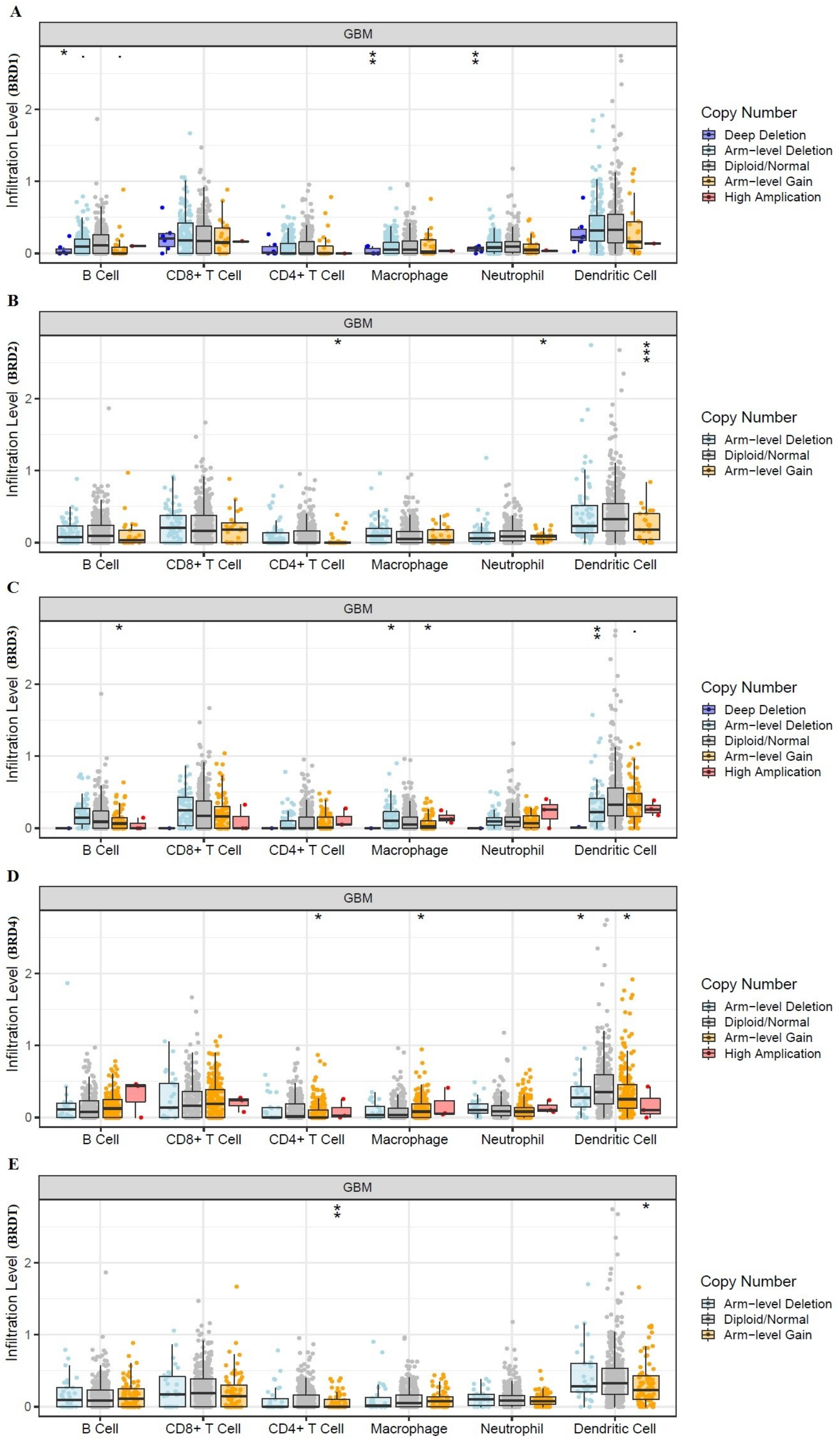

FIGURE 6. Effect of gene copy number changes expressed by different BET genes expression on immune cell infiltration in TIMER database. **(A)** BRD1, **(B)** BRD2, **(C)** BRD3 ,**(D)** BRD4 ,**(E)** BRDT.

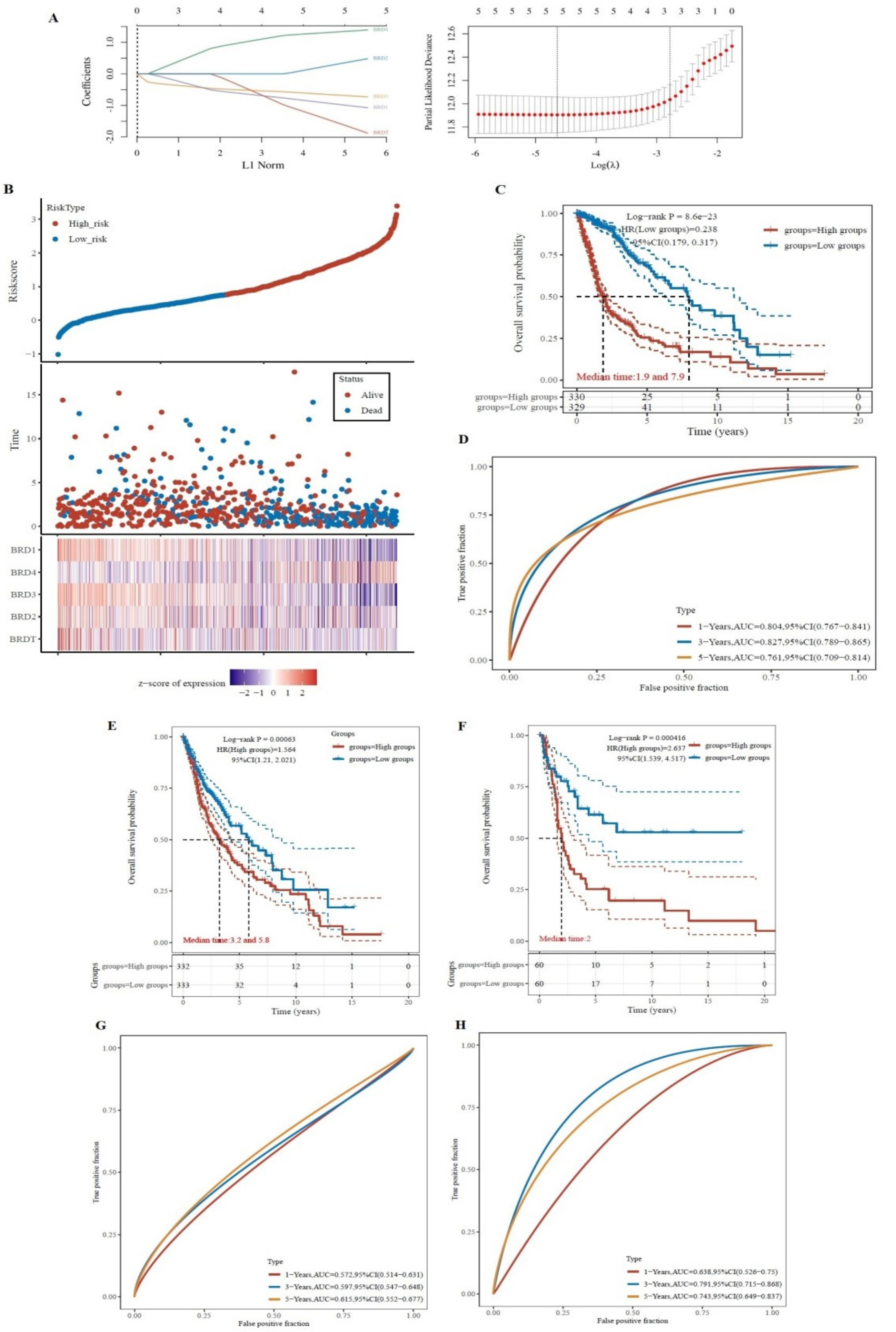

FIGURE 7. Analysis of prognostic assessment of BET genes in TCGA dataset(A-D), and external validation of BRD4 gene signature in ICGC training set(E-H). Patients were divided into low-risk and high-risk groups. **(A)** LASSO regression analysis was

performed on BET genes to calculate the correlation coefficients. Coefficients of selected features are shown by lambda parameter; Partial likelihood deviance versus log (λ)was drawn using LASSO Cox regression model. (**B**) The LASSO algorithm was used to generate risk scores for the training cohort from TCGA. Relationship of BET genes expression with risk score, survival time, and survival status were shown in the training cohort. (**C**) Distribution of KM survival curves for differential expression of BET genes in the training cohort. (**D**) ROC curve and AUC of BET genes signature classification. (**E**) Kaplan-Meier survival analysis of patients in different risk groups from TCGA dataset. (**F**) Kaplan-Meier survival analysis of patients in different risk groups from ICGC training set. (**G**) The ROC curves for risk score in TCGA dataset. (**H**) The ROC curves for risk score in ICGC training set. The higher values of AUC corresponding to higher predictive power.

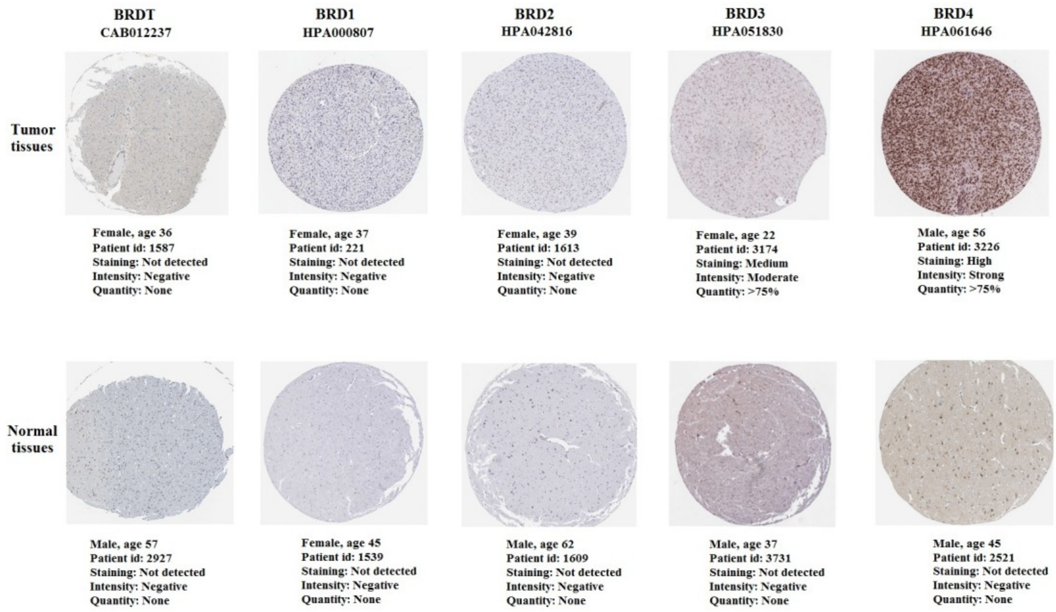

FIGURE 8. The immunohistochemistry of BET proteins in tumor tissues and normal cerebral cortex tissues(Scale bar: 200μm). The expression of BRD4 protein in tumor tissues was higher than that in normal tissues.

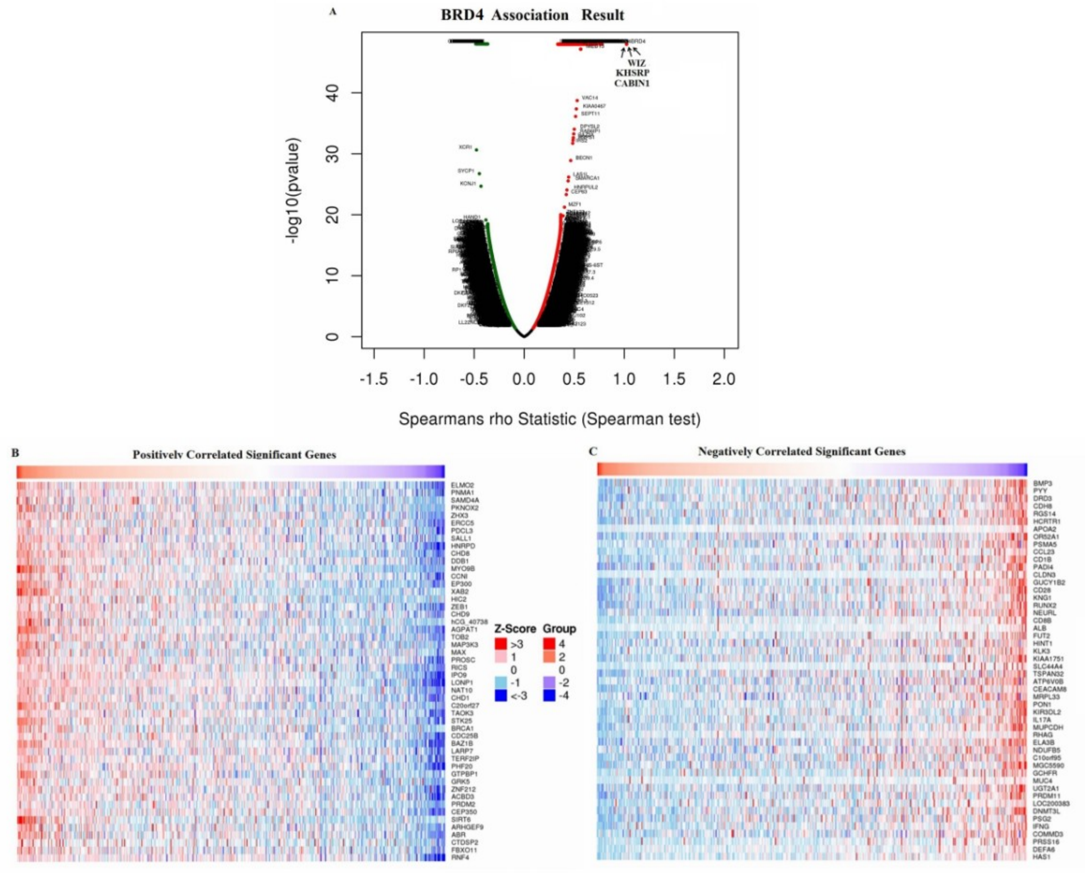

FIGURE 9. Differentially expressed genes associated with BRD4 in GBM samples in LinkedOmics database. **(A)** The correlation between BRD4 and differentially expressed genes in GBM was analyzed by Spearman test. **(B–C)** Heatmaps showed the positive and negative correlation genes (top 50) with BRD4 in GBM.

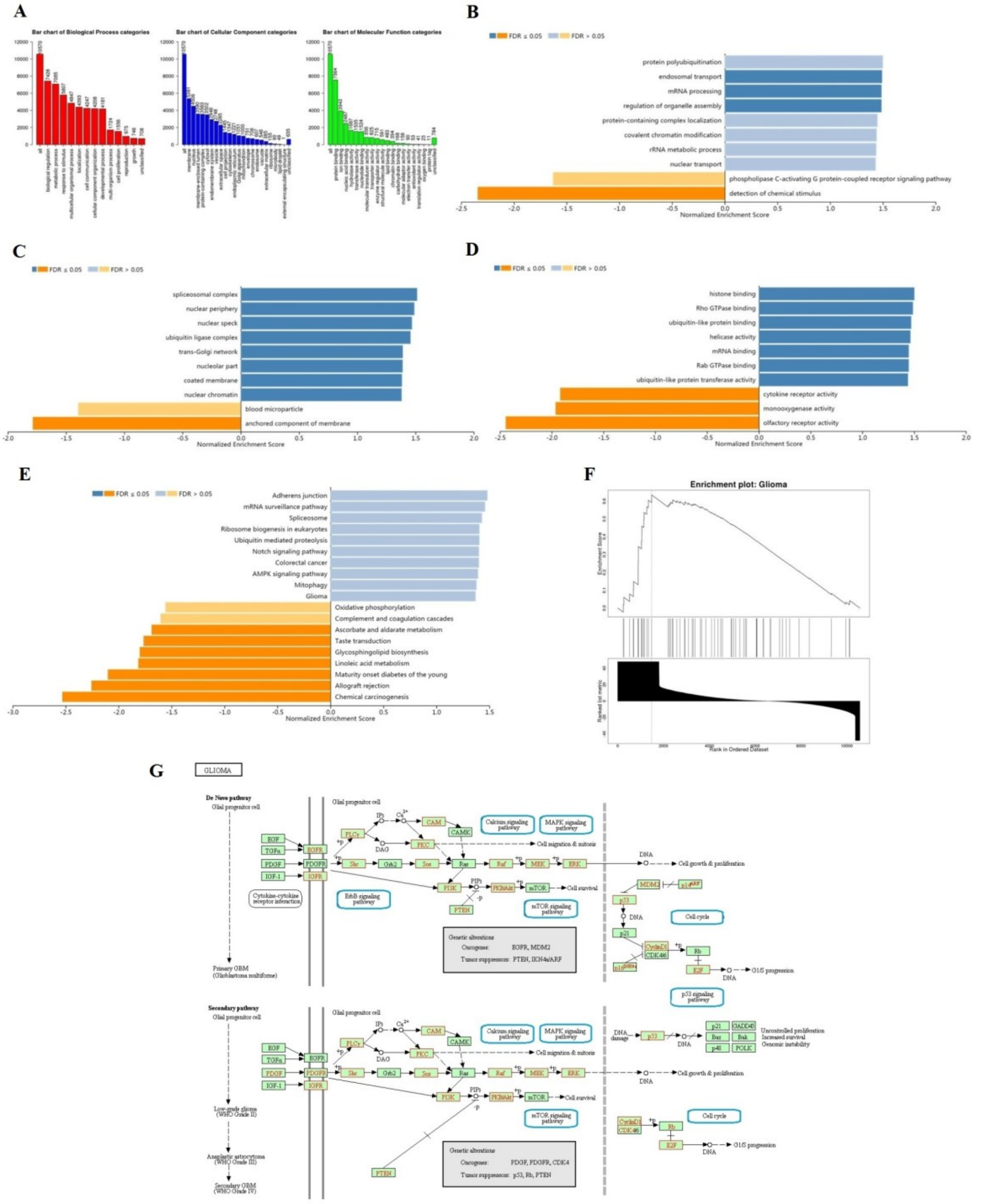

FIGURE 10.The function of BRD4 gene was predicted by the analysis of GO and KEGG in GSEA. **(A)** The GO enrichment analysis of target genes was predicted from three aspects: biological process (BP), cellular component (CC) and molecular function (MF). **(B)** The top 10 functional roles of BP for BRD4. **(C)** The top 10 functional roles of CC for BRD4. **(D)** The top 10 functional roles of MF for BRD4. **(E)** KEGG analysis of differentially expressed genes. **(F)** Enrichment analysis of KEGG pathway showed the regulation pathway of Glioma. **(G)** KEGG annotations of the

Glioma pathway regulated by BRD4 in brain cancer (cBioPortal). Targets marked in red were related to the Leading Edge Gene.

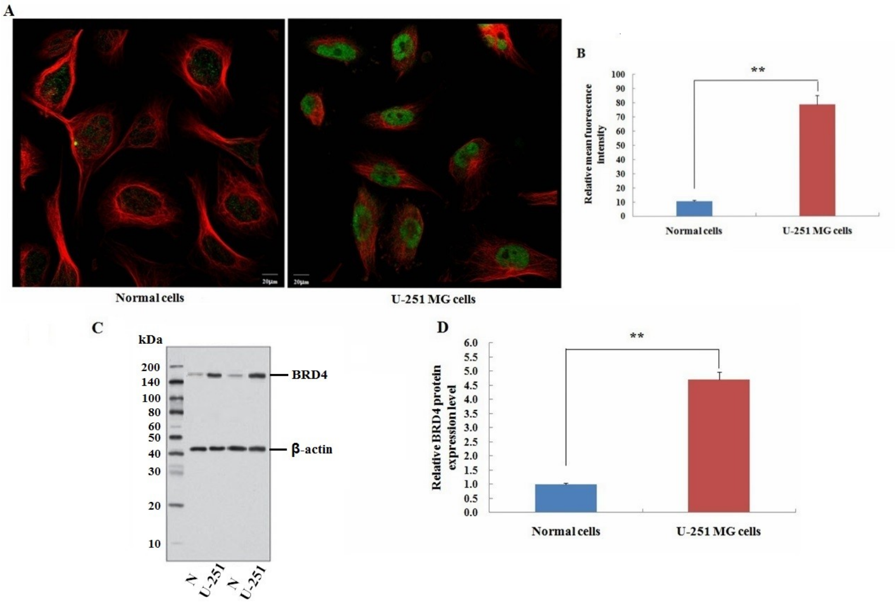

FIGURE 11. Validation experiment of BRD4 expression in cells. **(A)** Immunofluorescence analysis of BRD4 (green) in normal neuroglial cells and U-251 MG cells (Scale bar: 20 μm). **(B)** Mean relative fluorescence intensity values for each group. **(C)** Western blot analysis of BRD4 protein between normal neuroglial cells and U-251 MG cells. β-actin was an internal control. **(D)** Relative expression value of BRD4 protein (means ± SD, n = 3,**p < 0.01) .

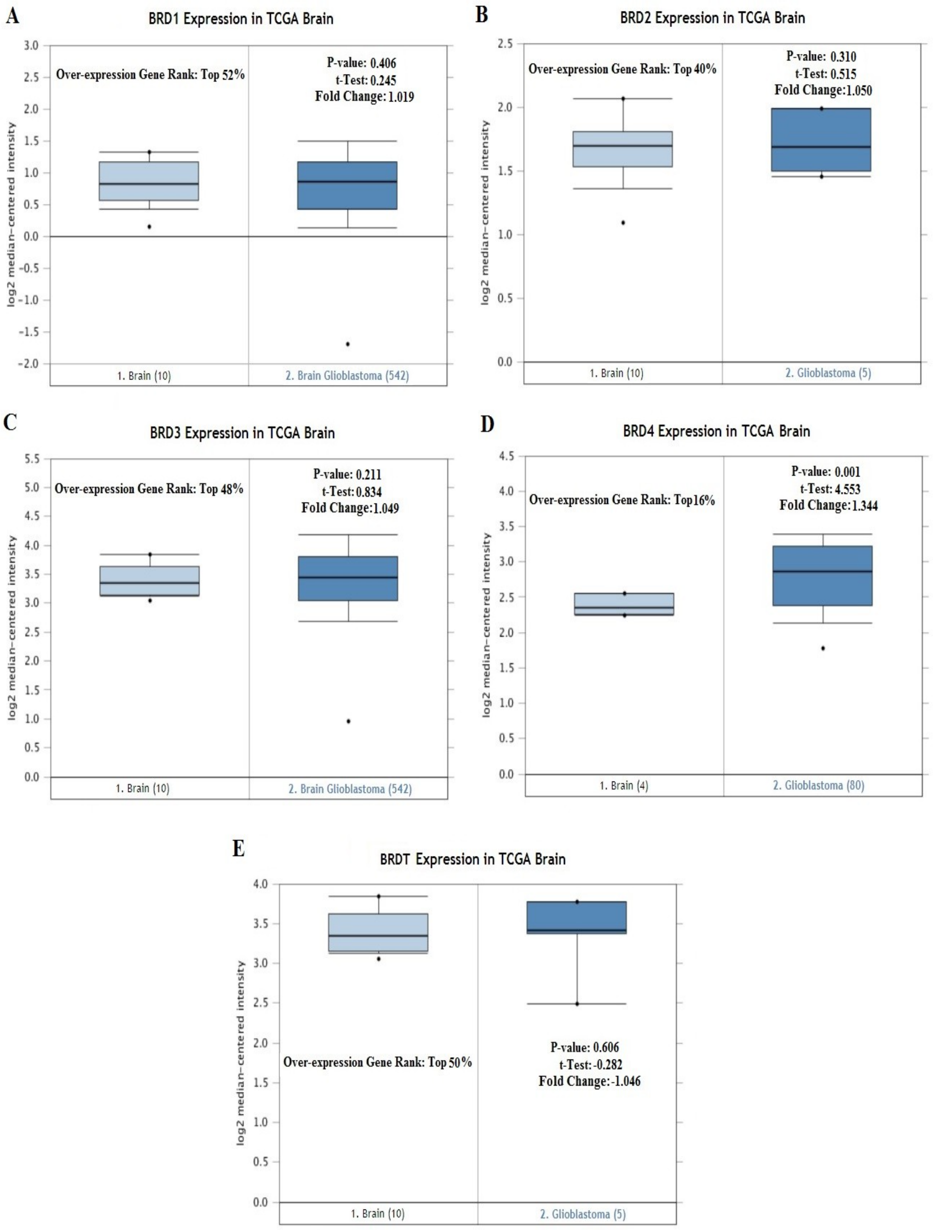

FIGURE S1. The transcription levels of BET genes in GBM samples. **(A-E)** The expression of BRD1, BRD2, BRD3, BRD4, BRDT were shown in TCGA Brain cohort, respectively. The p-values, t-Test, fold change, and over- expression gene rank were based on Oncomine database.

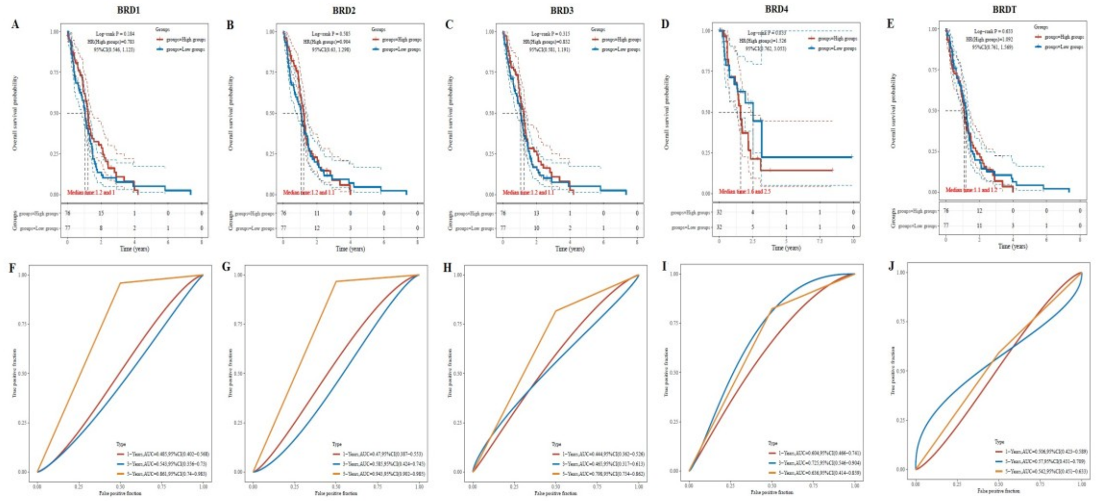

FIGURE S2. Analysis of prognostic assessment of BRD family member in TCGA dataset. **(A-E)** Kaplan-Meier survival analysis of BRD1, BRD2, BRD3 ,BRD4 and BRDT in different risk groups , respectively.**(F-J)** The ROC curves for risk score of BRD1, BRD2, BRD3 ,BRD4 and BRDT, respectively.

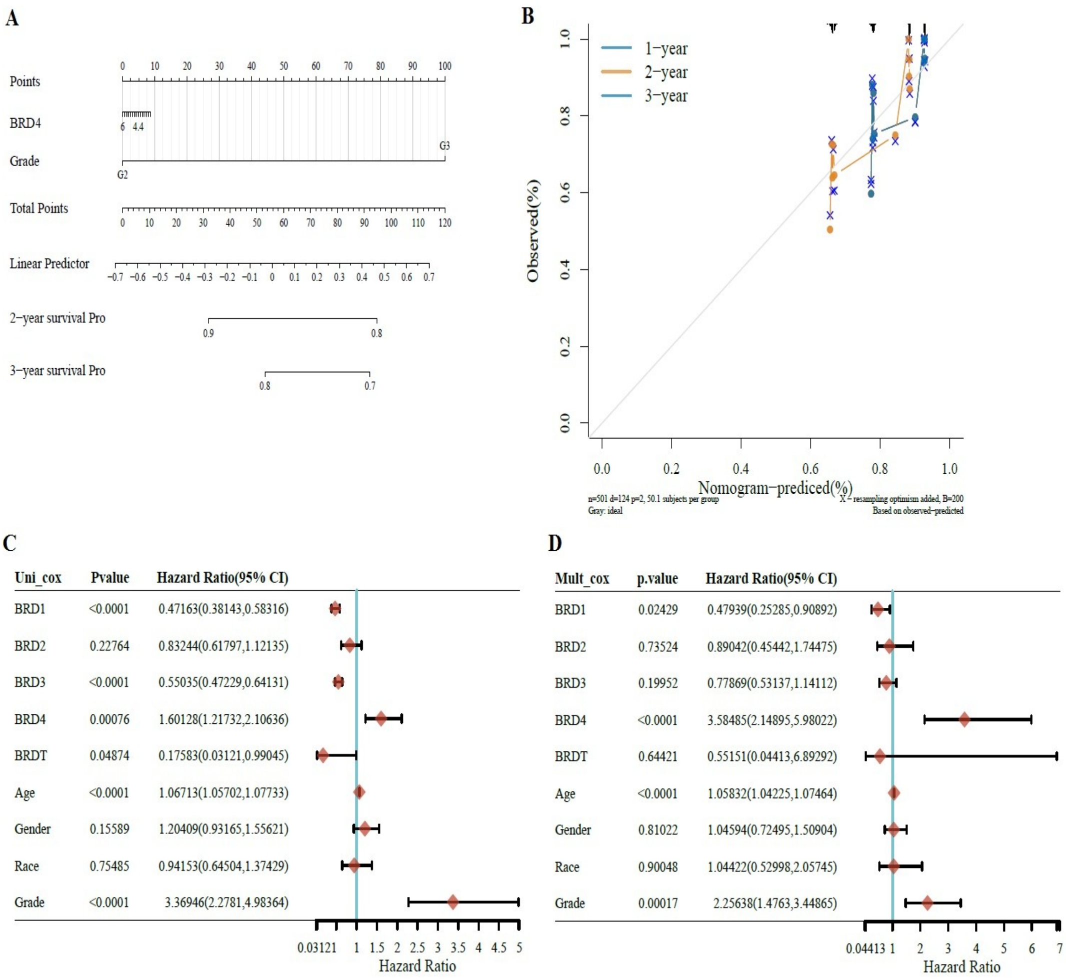

FIGURE S3. The nomogram was constructed by BET genes and the clinico-pathological characteristics in the cohort of TCGA-GBM. **(A)** The nomogram predicted the 2- and 3-year overall survival of GBM patients. **(B)** Calibration curves for the overall survival nomogram model in discovery group. **(C)** The risk coefficient and confidence interval were analyzed by univariate Cox regression. **(D)** The risk coefficient and confidence interval were analyzed by multivariate Cox regression.

TABLE

TABLE 1. The top 10 significant genes correlated with differentially expressed BET genes in GBM (GEPIA)

Gene	Correlated genes
BRD1	TTC28, ZBED4, PPP6R2, TCF20, PRR14L, MORC2, TNRC6B, NUP50, ZNF70, GTPBP1
BRD2	TRIM26,DHX16,ABCF1,SAFB,RBM10,LEMD2,COPS7B,TRIM39,UBR2,DVL3
BRD3	GTF3C4,CAMSAP1,EHMT1,PRRC2B,PRDM10,GATAD2B,FAM168B,PHF2,RC3H2,PBRM1
BRD4	WIZ,AP3D1,ARHGEF18,NACC1,KHSRP,GATAD2A,CHERP,TNPO2,SAFB,MAU2
BRDT	NUPL1P1,CRYGC,CTA-150C2.13,RP11-1281K21.8,RP5-1185K9.1,CTC-420A11.2,AMELX,RP11-532F6.2,RNA5SP218,RP11-1267H10.1
