# Supplementary material for: Comprehensive analysis of the prognosis and immune infiltrates for the BET protein family reveals the significance of BRD4 in glioblastoma multiforme
Source: Front Cell Dev Biol. 2023 Jan 12;11:1042490. doi: 10.3389/fcell.2023.1042490 (PMC9878708; doi:10.3389/fcell.2023.1042490)
Supplement: Supplementary file 4 [file DataSheet2.ZIP › Supplementary Material/Supplementary Table/Supplementary Table.docx]

**TABLES**

**TABLE 1.**Disease-gene association enrichment analysis of BET genes in DisGeNET (Metascape)

| **GO** | **Description** | **Count** | **(%)** | **Log10 (P)** | **Log10 (q)** |
| --- | --- | --- | --- | --- | --- |
| C0205833 | Medullomyoblastoma | 3 | 60 | -7.60 | -3.40 |
| C1275668 | Melanotic medulloblastoma | 3 | 60 | -7.60 | -3.40 |
| C0751291 | Desmoplastic Medulloblastoma | 3 | 60 | -7.30 | -3.20 |
| C0278701 | Gastric Adenocarcinoma | 3 | 60 | -4.50 | -0.57 |
| C0278876 | Adult Medulloblastoma | 3 | 60 | -3.80 | -0.06 |
| C0278510 | Childhood Medulloblastoma | 3 | 60 | -3.80 | -0.06 |

**Note:** "Log10(P)" is the p-value in log base 10. "Log10(q)" is the multi-test adjusted p-value in log base10.

**TABLE 2.** GO enrichment items of the differentiated BET genes (Metascape)

| **GO** | **Category** | **Description** | **Count** | **(%)** | **Log10 (P)** | **Log10 (q)** |
| --- | --- | --- | --- | --- | --- | --- |
| GO:0006325 | GO Biological Processes | chromatin organization | 5 | 100.00 | -8.47 | -4.13 |
| GO:0006338 | GO Biological Processes | chromatin remodeling | 4 | 80.00 | -7.34 | -3.30 |

**Note:** "Log10(P)" is the p-value in log base 10. "Log10(q)" is the multi-test adjusted p-value in log base10.

**TABLE 3**. The top 10 gene expression correlation analysis for BRD4 (LinkedOmics)

| **Target Gene/Attribute** | **Spearman Correlation** | **P-value** | **FDR** | **Event_SD** |
| --- | --- | --- | --- | --- |
| BRD4 | 1.000 | 1.000e-48 | 1.000e-47 | 528 |
| WIZ | 0.7719 | 1.000e-48 | 1.000e-47 | 528 |
| KHSRP | 0.7555 | 1.000e-48 | 1.000e-47 | 528 |
| CABIN1 | 0.7244 | 1.000e-48 | 1.000e-47 | 528 |
| GNA11 | 0.7237 | 1.000e-48 | 1.000e-47 | 528 |
| SMARCA4 | 0.7235 | 1.000e-48 | 1.000e-47 | 528 |
| KIAA0515 | 0.7208 | 1.000e-48 | 1.000e-47 | 528 |
| C19orf29 | 0.7118 | 1.000e-48 | 1.000e-47 | 528 |
| GATAD2A | 0.7087 | 1.000e-48 | 1.000e-47 | 528 |
| PIP5K1C | 0.7044 | 1.000e-48 | 1.000e-47 | 528 |

**TABLE** **4**. The top 10 functional roles of biological processes for BRD4

| **Gene Set** | **Description** | **Leading Edge Number** | **Normalized Enrichment Score(NES)** | **P Value** | **FDR** |
| --- | --- | --- | --- | --- | --- |
| GO:0030705 | cytoskeleton-dependent intracellular transport | 49 | 1.5000 | 0 | 0.056516 |
| GO:0006397 | mRNA processing | 132 | 1.4911 | 0 | 0.040821 |
| GO:0016197 | endosomal transport | 59 | 1.4813 | 0 | 0.039064 |
| GO:1902115 | regulation of organelle assembly | 58 | 1.4792 | 0 | 0.038966 |
| GO:0016072 | rRNA metabolic process | 69 | 1.4379 | 0 | 0.065012 |
| GO:0016569 | covalent chromatin modification | 124 | 1.4365 | 0 | 0.063479 |
| GO:0051169 | nuclear transport | 108 | 1.4298 | 0 | 0.064635 |
| GO:0010608 | posttranscriptional regulation of gene expression | 123 | 1.4030 | 0 | 0.064074 |
| GO:0002526 | acute inflammatory response | 48 | -1.6802 | 0 | 0.063072 |
| GO:0009593 | detection of chemical stimulus | 56 | -2.3212 | 0 | 0 |

**TABLE** **5**. The top 10 functional roles of cellular components(CC) for BRD4

| **Gene Set** | **Description** | **Leading Edge Number** | **Normalized Enrichment Score(NES)** | **P Value** | **FDR** |
| --- | --- | --- | --- | --- | --- |
| GO:0005681 | spliceosomal complex | 53 | 1.5144 | 0 | 0.011015 |
| GO:0034399 | nuclear periphery | 54 | 1.4904 | 0 | 0.016890 |
| GO:0016607 | nuclear speck | 118 | 1.4700 | 0 | 0.017951 |
| GO:0000151 | ubiquitin ligase complex | 65 | 1.4575 | 0 | 0.019736 |
| GO:0005802 | trans-Golgi network | 51 | 1.3911 | 0 | 0.033182 |
| GO:0044452 | nucleolar part | 38 | 1.3902 | 0 | 0.031273 |
| GO:0048475 | coated membrane | 28 | 1.3838 | 0 | 0.032868 |
| GO:0000790 | nuclear chromatin | 94 | 1.3810 | 0 | 0.033161 |
| GO:0072562 | blood microparticle | 28 | -1.3956 | 0 | 0.14986 |
| GO:0031225 | anchored component of membrane | 48 | -1.7850 | 0 | 0.028342 |

**TABLE** **6**.The top 10 functional roles of molecular functions(MF) for BRD4

| **Gene Set** | **Description** | **Leading Edge Number** | **Normalized Enrichment Score(NES)** | **P Value** | **FDR** |
| --- | --- | --- | --- | --- | --- |
| GO:0042393 | histone binding | 64 | 1.5023 | 0 | 0.019699 |
| GO:0017048 | Rho GTPase binding | 46 | 1.4887 | 0 | 0.018005 |
| GO:0032182 | ubiquitin-like protein binding | 38 | 1.4721 | 0 | 0.022238 |
| GO:0004386 | helicase activity | 59 | 1.4646 | 0 | 0.022860 |
| GO:0017137 | Rab GTPase binding | 42 | 1.4485 | 0 | 0.026992 |
| GO:0003729 | mRNA binding | 67 | 1.4485 | 0 | 0.028413 |
| GO:0019787 | ubiquitin-like protein transferase activity | 94 | 1.4405 | 0 | 0.028958 |
| GO:0004896 | cytokine receptor activity | 28 | -1.9179 | 0 | 0.021093 |
| GO:0004497 | monooxygenase activity | 39 | -1.9626 | 0 | 0.017718 |
| GO:0004984 | olfactory receptor activity | 32 | -2.4443 | 0 | 0 |

**TABLE** **7**.The top 19 KEGG pathway analysis for BRD4

| **Gene Set** | **Description** | **Leading Edge Number** | **Normalized Enrichment Score(NES)** | **P Value** | **FDR** |
| --- | --- | --- | --- | --- | --- |
| hsa04520 | Adherens junction | 32 | 1.4821 | 0 | 0.085886 |
| hsa03015 | mRNA surveillance pathway | 35 | 1.461 | 0 | 0.08834 |
| hsa03040 | Spliceosome | 41 | 1.4313 | 0 | 0.10049 |
| hsa03008 | Ribosome biogenesis in eukaryotes | 20 | 1.4071 | 0 | 0.10217 |
| hsa04120 | Ubiquitin mediated proteolysis | 51 | 1.4064 | 0 | 0.094338 |
| hsa04330 | Notch signaling pathway | 13 | 1.4031 | 0.010121 | 0.081687 |
| hsa05210 | Colorectal cancer | 24 | 1.4025 | 0.0020121 | 0.077502 |
| hsa04152 | AMPK signaling pathway | 36 | 1.3946 | 0 | 0.081523 |
| hsa04137 | Mitophagy | 23 | 1.3797 | 0.010142 | 0.098973 |
| hsa05214 | Glioma | 18 | 1.371 | 0.0040241 | 0.097678 |
| hsa00190 | Oxidative phosphorylation | 29 | -1.5586 | 0 | 0.089424 |
| hsa04610 | Complement and coagulation cascades | 24 | -1.6045 | 0 | 0.068344 |
| hsa00053 | Ascorbate and aldarate metabolism | 3 | -1.6884 | 0 | 0.040316 |
| hsa04742 | Taste transduction | 27 | -1.7642 | 0 | 0.030055 |
| hsa00601 | Glycosphingolipid biosynthesis | 11 | -1.8008 | 0 | 0.0243 |
| hsa00591 | Linoleic acid metabolism | 8 | -1.8143 | 0.022222 | 0.021441 |
| hsa04950 | Maturity onset diabetes of the young | 9 | -2.1016 | 0 | 0 |
| hsa05330 | Allograft rejection | 14 | -2.2568 | 0 | 0 |
| hsa05204 | Chemical carcinogenesis | 22 | -2.5317 | 0 | 0 |
