# Supplementary material for: Comprehensive analysis of the prognosis and immune infiltrates for the BET protein family reveals the significance of BRD4 in glioblastoma multiforme
Source: Front Cell Dev Biol. 2023 Jan 12;11:1042490. doi: 10.3389/fcell.2023.1042490 (PMC9878708; doi:10.3389/fcell.2023.1042490)
Supplement: Supplementary file 4 [file DataSheet2.ZIP › Supplementary Material/Supplementary Image/Supplementary Image.doc]

**Supplementary Image**

**FIGURE S1**. The transcription levels of BET genes in GBM samples. **(A-E)** The expression of BRD1, BRD2, BRD3, BRD4, BRDT were shown in TCGA Brain cohort, respectively. The p-values, t-Test, fold change, and over- expression gene rank were based on Oncomine database.

**FIGURE S2**. Analysis of prognostic assessment of BRD family member in TCGA dataset.  **(A-E)** Kaplan-Meier survival analysis of BRD1, BRD2, BRD3 ,BRD4 and BRDT in different risk groups , respectively.**(F-J)** The ROC curves for risk score of BRD1, BRD2, BRD3 ,BRD4 and BRDT, respectively.

**FIGURE S3 .** The nomogram was constructed by BRDs and the clinicopathological characteristics in the cohort of TCGA-GBM . **(A)** The nomogram predict the 2- and 3-year overall survival of GBM patients. **(B)** Calibration curves for the overall survival nomogram model in discovery group. **(C)** The risk coefficient and confidence interval were analyzed by univariate Cox regression. **(D)** The risk coefficient and confidence interval were analyzed by multivariate Cox regression.

.
